# Stratification and therapeutic potential of PML in metastatic breast cancer

**DOI:** 10.1038/ncomms12595

**Published:** 2016-08-24

**Authors:** Natalia Martín-Martín, Marco Piva, Jelena Urosevic, Paula Aldaz, James D. Sutherland, Sonia Fernández-Ruiz, Leire Arreal, Verónica Torrano, Ana R. Cortazar, Evarist Planet, Marc Guiu, Nina Radosevic-Robin, Stephane Garcia, Iratxe Macías, Fernando Salvador, Giacomo Domenici, Oscar M. Rueda, Amaia Zabala-Letona, Amaia Arruabarrena-Aristorena, Patricia Zúñiga-García, Alfredo Caro-Maldonado, Lorea Valcárcel-Jiménez, Pilar Sánchez-Mosquera, Marta Varela-Rey, Maria Luz Martínez-Chantar, Juan Anguita, Yasir H. Ibrahim, Maurizio Scaltriti, Charles H. Lawrie, Ana M. Aransay, Juan L. Iovanna, Jose Baselga, Carlos Caldas, Rosa Barrio, Violeta Serra, Maria dM Vivanco, Ander Matheu, Roger R. Gomis, Arkaitz Carracedo

**Affiliations:** 1 CIC bioGUNE, Bizkaia Technology Park, Bulding 801a, 48160 Derio, Spain; 2 Oncology Programme, Institute for Research in Biomedicine (IRB-Barcelona), 08028 Barcelona, Spain; 3 Oncology Area, Biodonostia Institute, 20014 San Sebastian, Spain; 4 Biostatistics and Bioinformatics Unit, Institute for Research in Biomedicine (IRB-Barcelona), 08028 Barcelona, Spain; 5 School of Life Sciences, Ecole Polytechnique Fédérale de Lausanne (EPFL), 1015 Lausanne, Switzerland; 6 ERTICa Research Group, University of Auvergne EA4677, Clermont-Ferrand, France; 7 Biodiagnostics Laboratory OncoGenAuvergne, Pathology Unit, Jean Perrin Comprehensive Cancer Center, 63000 Clermont-Ferrand, France; 8 Centre de Recherche en Cancérologie de Marseille (CRCM), INSERM UMR 1068, CNRS UMR 7258, Aix-Marseille University and Institut Paoli-Calmettes, Parc Scientifique et Technologique de Luminy, 13288 Marseille, France; 9 Cancer Research UK Cambridge Institute, University of Cambridge, Li Ka Shing Centre, Robinson Way, Cambridge CB2 0RE, UK; 10 Centro de Investigación Biomédica en Red de Enfermedades Hepáticas y Digestivas (CIBERehd); 11 IKERBASQUE, Basque foundation for science, 48013 Bilbao, Spain; 12 Experimental Therapeutics Group, Vall d'Hebron University Hospital, 08035 Barcelona, Spain; 13 Weill Cornell Medicine, New York 10021, USA; 14 Human Oncology and Pathogenesis Program, Department of Pathology, Memorial Sloan-Kettering Cancer Center, 10065 New York, USA; 15 Human Oncology and Pathogenesis Program, Department of Medicine, Memorial Sloan-Kettering Cancer Center, 10065 New York, USA; 16 Institució Catalana de Recerca i Estudis Avançats (ICREA), 08010 Barcelona, Spain; 17 Biochemistry and Molecular Biology Department, University of the Basque Country (UPV/EHU), 48949 Leioa, Spain

## Abstract

Patient stratification has been instrumental for the success of targeted therapies in breast cancer. However, the molecular basis of metastatic breast cancer and its therapeutic vulnerabilities remain poorly understood. Here we show that PML is a novel target in aggressive breast cancer. The acquisition of aggressiveness and metastatic features in breast tumours is accompanied by the elevated PML expression and enhanced sensitivity to its inhibition. Interestingly, we find that STAT3 is responsible, at least in part, for the transcriptional upregulation of PML in breast cancer. Moreover, PML targeting hampers breast cancer initiation and metastatic seeding. Mechanistically, this biological activity relies on the regulation of the stem cell gene *SOX9* through interaction of PML with its promoter region. Altogether, we identify a novel pathway sustaining breast cancer aggressiveness that can be therapeutically exploited in combination with PML-based stratification.

## Abstract (web summary)

Targeting PML in acute promyelocytic leukaemia has changed the outcome of patients with this disease. Here, the authors demonstrated that PML is also a potential therapeutic target in breast cancer where it specifically regulates cancer initiating cells and tumour progression through the transcriptional regulation of *SOX9*.

## Graphical abstract

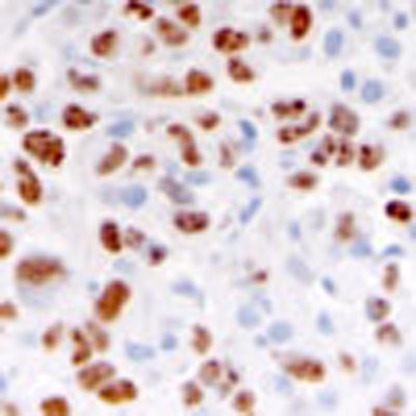

Patient stratification for cancer therapy is an excellent illustration of precision medicine, and biomarker-based treatment selection has tremendously aided in the success of current cancer therapies^1^. In this sense, the current ability to molecularly define and differentiate breast cancer (BCa) into molecular subtypes^2^,^3^ has allowed the identification of patients at risk of relapse^4^ and has led to biomarker signatures used to spare low-risk patients from aggressive chemotherapy^5^.

Tumours are heterogeneous entities and most cancers retain a differential fraction of cells with increased self-renewal capability (cancer stem or initiating cells)^6^. Cancer-initiating cells (CICs) exhibit a unique spectrum of biological, biochemical and molecular features that have granted them an important role in disease recurrence and metastatic dissemination in BCa^7^,^8^. Despite the accepted relevance of CICs in cancer progression, the molecular cues governing their activity and function remain largely unknown. The sex determining region Y Box 9 (SOX9) is a recently described regulator of cell differentiation and self-renewal^9^,^10^,^11^ and is found upregulated in BCa^12^,^13^,^14^.

The promyelocytic leukaemia (PML) protein negatively regulates survival and proliferation pathways in cancer, functions that have established it as a classical pro-apoptotic and growth inhibitory tumour suppressor^15^,^16^. PML is the essential component of multi-protein sub-nuclear structures commonly referred to as the PML nuclear bodies. PML multimerizes to function as a scaffold critical for the composition and assembly of the entire complex, a process that is regulated by Small Ubiquitin-like Modifier (SUMO)-mediated modifications and interactions^15^,^16^. Despite the general perception of being PML a bona fide tumour suppressor in cancer, a series of recent studies have demonstrated that PML exhibits activities in cancer that go far and beyond tumour suppression^17^. The work in chronic myeloid leukaemia has evidenced that PML expression can be promoted in certain cancers, providing a selective advantage to tumour cells^18^,^19^. Moreover, PML is found upregulated in a subset of BCa^20^. However, to which extent PML targeting could be a valuable therapeutic approach in solid cancers remains obscure.

In this study, we reveal the therapeutic and stratification potential of PML in BCa and the molecular cues, underlying the therapeutic response unleashed by PML inhibition.

## Results

### PML silencing hampers BCa-initiating cell capacity

**Figure 1:**
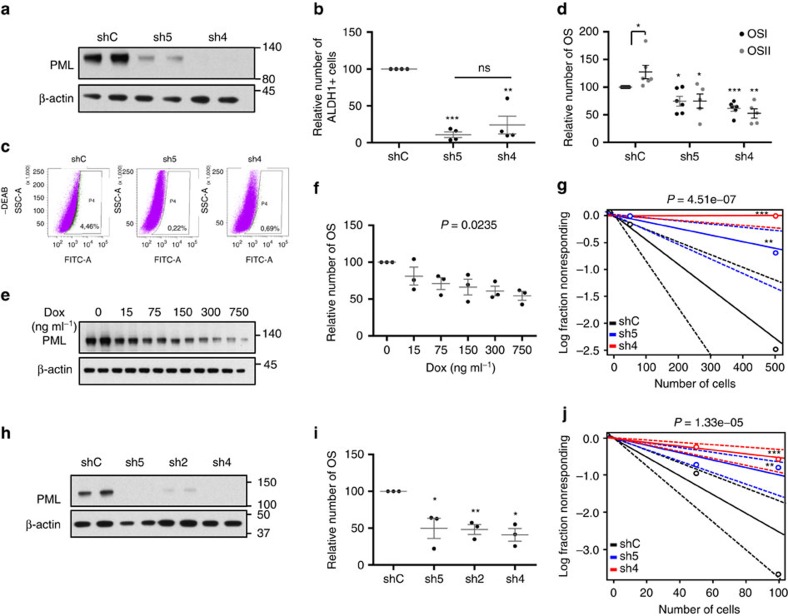
Genetic targeting of *PML* hampers breast cancer initiation potential. (**a**) PML levels (representative western blot out of four independent experiments) upon *PML* silencing with two shRNAs (sh) in MDA-MB-231 cells. (**b**) Percentage of ALDH1+ cells upon *PML* silencing with two shRNAs in MDA-MB-231 cells (*n*=4). (**c**) Representative flow cytometry analysis out of three independent experiments of the ALDH1+ population in shC or shPML-transduced MDA-MB-231 cells (FITC: fluorescein-isothiocyanate, SSC-A: side-scatter). (**d**) Effect of *PML* silencing on primary (OSI) and secondary (OSII) OS formation in MDA-MB-231 cells (*n*=5 for OSII in shPML cells and *n*=6 for shC and OSI in shPML cells). (**e**,**f**) PML levels (representative western blot out of three independent experiments) (**e**) and OS formation (*n*=3) (**f**) upon *PML* inducible silencing (shPML#4) with the indicated doses of doxycycline in MDA-MB-231 cells. (**g**) Limiting dilution experiment after xenotransplantation. Nude mice were inoculated with 500,000 or 50,000 MDA-MB-231 cells (*n*=12 injections per experimental condition). Tumour-initiating cell number was calculated using the ELDA platform. A log-fraction plot of the limiting dilution model fitted to the data is presented. The slope of the line is the log-active cell fraction (solid lines: mean; dotted lines: 95% confidence interval; circles: values obtained in each cell dilution). (**h**) PML levels (representative western blot out of four independent experiments) upon *PML* silencing in the PDX44-derived cell line. (**i**) OSI formation upon *PML* silencing in PDX44 cells (*n*=3). (**j**) Limiting dilution experiment after xenotransplantation. Nude mice were inoculated either with 100,000 or 10,000 PDX44 cells (*n*=20 injections per experimental condition). Tumour-initiating cell number was calculated using the ELDA platform as in **g**. Error bars represent s.e.m., *P* value (*P<0.05; **P<0.01; ***P<0.001 compared with shC or as indicated). Statistics test: one-tail unpaired *t-*test (**b**,**d**,**i**), analysis of variance (**f**) and *χ*^2^-test (**g**,**j**). dox, doxycycline; OS, oncospheres; shC, Scramble shRNA; sh2, sh4 and sh5, shRNA against *PML*.

The elevated expression of PML in a subset of BCa^17^,^20^ strongly suggests that it could represent an attractive target for therapy. To ascertain the molecular and biological processes controlled by PML in BCa, we carried out short hairpin RNA (shRNA) lentiviral delivery-mediated *PML* silencing in different cellular systems. Four constitutively expressed shRNAs exhibited activity against *PML* (Fig. 1a; Supplementary Fig. 1a–d). *PML* knockdown elicited a potent reduction in the number of ALDH1-positive cells and in oncosphere formation (OS, readout of self-renewal potential^7^,^21^), in up to three PML-high-expressing basal-like BCa (BT549 and MDA-MB-231) or immortalized (HBL100) cell lines tested (Fig. 1b–d; Supplementary Fig. 1e–g). This phenotype was recapitulated with a doxycycline-inducible lentiviral shRNA system targeting *PML* (sh4; Fig. 1e,f; Supplementary Fig. 1h).

Self-renewal capacity is a core feature of CICs^7^. On the basis of this notion, we hypothesized that PML could regulate tumour initiation in BCa. We performed tumour formation assays in immunocompromised mice, using MDA-MB-231 cells (PML-high-expressing triple-negative breast cancer (TNBC)) transduced with non-targeting (shRNA Scramble: shC) or *PML*-targeting shRNAs. *PML* silencing exhibited a profound defect in tumour formation capacity, resulting in a decrease in the frequency of tumour-initiating cells from 1/218 (shC) to 1/825 (sh5) and completely abolished (1/infinite) in sh4 (Fig. 1g; Supplementary Fig. 1i).

To extrapolate these observations to the complexity of human BCa, we characterized a series of patient-derived xenografts (PDXs; Table 1; Supplementary Fig. 1j). The distribution of PML expression in the different subtypes of engrafted tumours was reminiscent of patient data, with a higher proportion of PML-high-expressing tumours in basal-like/triple-negative subtype^20^. Taking advantage of the establishment of a PML-high-expressing PDX-derived cell line (PDX44), we sought to corroborate the results obtained in the PML-high-expressing cell lines. As with MDA-MB-231 cells, *PML* silencing was effective in the PDX44-derived cell line (Fig. 1h) and resulted in a significant decrease in OS formation (Fig. 1i). *In vivo*, *PML* silencing decreased tumour-forming capacity of PDX44 cells (tumour-initiating cell frequency was estimated of 1/39.6 in shC, 1/100 in sh5 and 1/185 in sh4; Fig. 1j; Supplementary Fig. 1k).

These data demonstrate that PML expression is required for BCa-initiating cell function in TNBC cells.

### PML sustains metastatic potential in BCa

**Figure 2:**
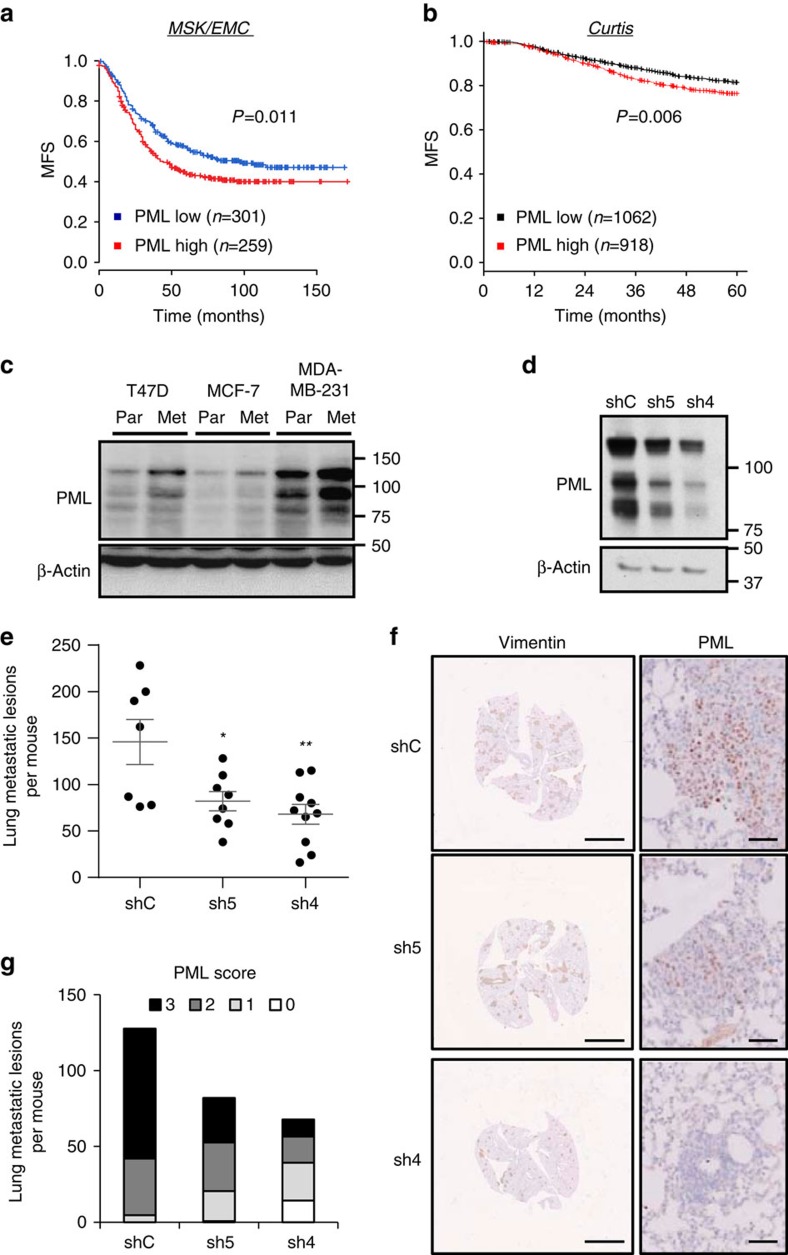
PML is associated to breast cancer metastatic dissemination. (**a**,**b**) Kaplan–Meier representations of MFS based on *PML* RNA expression. (**a**) MSK/EMC data set, *n*=560. (**b**) Curtis data set (MFS before 60 months), *n*=1980. PML high: above the mean expression; PML low: below the mean expression. (**c**) Representative western blot out of three independent experiments, showing PML protein expression in cell line sub-clones selected for high metastatic potential (Par=parental and Met=metastatic). (**d–g**) Effect of PML silencing on metastatic capacity of intravenously injected metastatic MDA-MB-231 sub-clones (*n*=10 mice per condition): Western blot showing PML silencing in cells at the time of injection (**d**), number of metastatic lesions (**e**), representative immunostaining of Vimentin (scale bar, 3 mm) and PML (scale bar, 50μm) as indicator of metastatic lesions (**f**), and number of metastatic lesions for each PML immunoreactivity score (22 metastatic foci were scored and extrapolated to the number of total metastatic foci in each lung) (**g**). Error bars represent s.e.m., *P* value (*P<0.05; **P<0.01 compared with shC). Statistical test: Gehan–Breslow–Wilcoxon test (**a**,**b**) and one-tail unpaired *t*-test (**e**). MFS, metastasis-free survival; shC: Scramble shRNA; sh4 and sh5, shRNA against *PML*.

CIC activity is associated with tumour initiation and recurrence^7^,^22^. We have previously shown that PML expression is associated to early recurrence^20^, which we validated in an independent data set^23^ (Supplementary Fig. 2a). The development of metastatic lesions is based on the acquisition of novel features by cancer cells^24^. On the basis of our data, we surmised that the activity of PML on CICs could impact on the survival and growth in distant organs. To test this hypothesis, we measured metastasis-free survival (MFS) in two well-annotated large messenger RNA (mRNA) data sets^3^,^25^,^26^. First, we evaluated the impact of high *PML* expression in MFS in the MSK/EMC (Memorial Sloan Kettering Cancer Center-Erasmus Medical Center) data set^25^,^26^. As predicted, *PML* expression above the mean was associated with reduced MFS (Fig. 2a). Second, we validated this observation in the METABRIC data set, focusing on early metastasis (up to 5 years)^3^. On the one hand, we confirmed the MSK/EMC data (Fig. 2b; hazard ratio (HR)=1.31, log-rank test *P*=0.006). On the other hand, a Cox continuous model demonstrated an association of PML expression with the increased risk of metastasis (HR=2.305, *P*=0.002). Of note, we tested the expression of PML in patients with complete pathological response or residual disease after therapy^27^, but could not find a significant association of these parameters in two data sets (Supplementary Fig. 2b).

The molecular alterations associated to metastatic capacity can be studied using BCa cell lines, in which metastatic cell sub-clones have been selected through the sequential enrichment in immunocompromised mice^28^. If PML is a causal event in the acquisition of metastatic capacity, then changes in its expression should be observed in this cellular system. As predicted, PML mRNA and protein expression were elevated in three distinct metastatic sub-clones compared with their parental counterparts^25^,^26^ (Fig. 2c; Supplementary Fig. 2c).

Metastasis surrogate assays provide valuable information about the capacity of cancer cells to home and colonize secondary organs^29^. TNBC cells exhibit metastatic tropism to the lung^30^, and the molecular requirements of this process have begun to be clarified through the generation of highly metastatic sub-clones^31^. Our patient analysis suggests that PML expression is favoured in primary tumours, with higher capacity to disseminate. Moreover, cell sub-clone analysis further reveals that PML expression is selected for in the process of metastatic selection. With this data in mind, we asked to which extent PML would be responsible for the enhanced metastatic capacity. To address this question, we silenced *PML* in a highly metastatic sub-clone derived from MDA-MB-231 and injected these cells in the tail vein of nude mice. We chose tail vein injection due to the fact that other metastasis models based on the orthotopic implantation of cells in the mammary fat pad^25^ are influenced by primary tumour formation, which we reported to be altered by PML (Fig. 1). The reduction of PML was confirmed in the injected cells (Fig. 2d). Strikingly, *PML* silencing led to a significant reduction in lung metastatic foci formation (Fig. 2e). When evaluating the immunoreactivity of PML in the metastatic lesions (Fig. 2e–g), we observed a direct association between PML silencing at the time of injection (Fig. 2d) and the immunoreactivity of PML in metastatic foci (Fig. 2g). We evaluated whether the lack of PML could be limiting metastatic growth capacity by eliciting an apoptotic response, rather than CIC capacity. However, no differential apoptosis was detected by the means of cleaved caspase-3 staining (Supplementary Fig. 2d–e).

These data demonstrate that the genetic targeting of *PML* results in a tumour-suppressive response, characterized by decreased BCa-initiating cell function and consequently, reduced tumour initiation and metastasis.

### STAT3 participates in the regulation of PML expression

**Figure 3:**
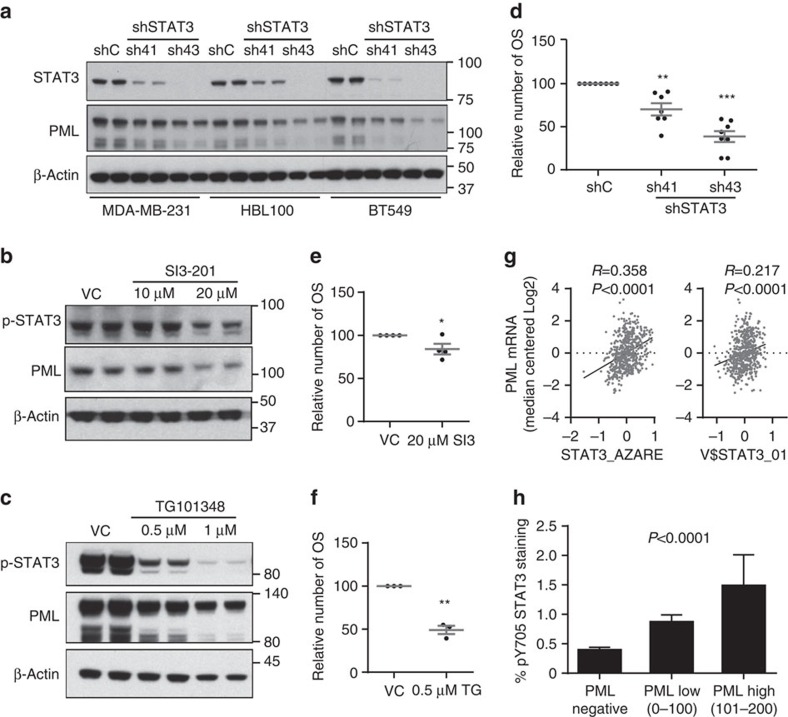
STAT3 regulates PML expression in breast cancer. (**a**) Representative western blot out of three independent experiments showing STAT3 and PML protein expression upon *STAT3* silencing with two different shRNA (sh41 and sh43). (**b**,**c**) Representative western blot out of three independent experiments, showing STAT3 and PML protein expression upon STAT3 inhibition using SI3-201 (**b**) and TG101314 (**c**) in MDA-MB-231 cells. (**d–f**) Effect of STAT3 inhibition on primary OS formation using sh41 and sh43 against *STAT3* (*n*=7) (**d**), SI3-201 (SI3; *n*=4) (**e**) and TG101314 (TG; *n*=3) (**f**) in MDA-MB-231 cells. (**g**) Correlation of two different STAT3 gene signatures with *PML* gene expression in the MSK/EMC data set. (**h**) Immunoreactivity of pY705 STAT3 protein in patient biopsies with varying expression of PML in the Marseille cohort (*n*=737). Error bars represent s.e.m., *P* value (*P<0.05; **P<0.01; ***P<0.001 compared with shC or VC as indicated). Statistics test: one-tail unpaired *t-*test (**d**,**e**,**f**), Pearson correlation (**g**) and analysis of variance (**h**). OSI, primary oncospheres; shC, Scramble shRNA; sh41 and sh43, shRNA against *STAT3*; VC, vehicle control.

Our data demonstrate that *PML* is transcriptionally regulated in BCa. *PML* gene expression is regulated upon various external stimuli, including type I and II interferons and interleukin 6, which are mediated by interferon regulatory factors and signal transducers and activators of transcription (STATs), respectively^32^,^33^,^34^,^35^. Specifically, it has been reported that activated STAT3 but not STAT1 correlates with *PML* mRNA and protein levels in fibroblasts, HeLa and U2OS cell lines^34^. Since, STAT3 is activated in oestrogen receptor (ER)-negative BCa^36^, we hypothesized that this transcription factor may be responsible for the transcriptional activation of *PML* in this tumour type. We silenced *STAT3* with two different short hairpins (sh41 and sh43), and showed that this approach led to the decrease in PML protein and gene expression in the different cell lines tested (Fig. 3a; Supplementary Fig. 3a–b). Moreover, pharmacological inhibition of the Janus kinase/signal transducers and activators of transcription (JAK/STAT) pathway at two different levels (SI3-201, an inhibitor of STAT3 phosphorylation and activation; TG1013148, a potent and highly selective ATP-competitive inhibitor of JAK2) decreased PML levels (Fig. 3b,c). In coherence with the activity of PML, genetic and pharmacological inhibition of STAT3 in MDA-MB-231 cells reduced the primary OS formation capacity (Fig. 3d–f). Importantly, *PML* gene expression levels in a cohort of 448 patients (MSK/EMC) correlated with the activity of STAT3, as confirmed with two different STAT3 signatures (Fig. 3g; ref. 37; http://software.broadinstitute.org/gsea/msigdb/cards/V$STAT3_01). In addition, immunohistochemical analysis confirmed an association between the PML immunoreactivity and phosphorylated STAT3 levels in the Marseille cohort (Fig. 3h). Our results provide strong support for the role of STAT3 as an upstream regulator of PML in BCa.

### Elevated PML expression predicts response to arsenic trioxide

**Figure 4:**
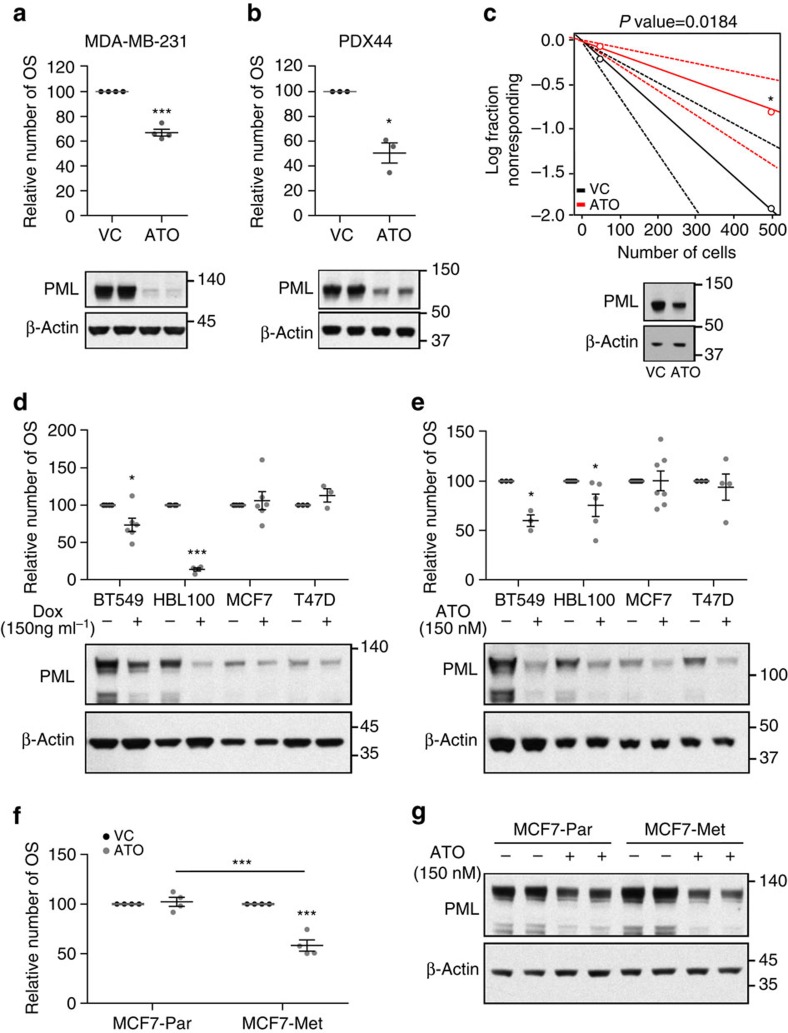
PML inhibition selectively targets PML-high-expressing breast cancer cells. (**a**,**b**) Effect of 150 nM ATO treatment on OSI formation (top panels) in MDA-MB-231 (*n*=4) (**a**) and PDX44 cells (*n*=3) (**b**) and PML protein expression (3-day treatment, lower panels, representative western blot out of four—MDA-MB-231—or three—PDX44—independent experiments). (**c**) Limiting dilution experiment after xenotransplantation. Nude mice were inoculated with 500,000 or 50,000 MDA-MB-231 cells (*n*=20 injections per experimental condition). ATO cells were pre-treated with 150 nM ATO 2 days before injection. Tumour-initiating cell number was calculated using the ELDA platform. A log-fraction plot of the limiting dilution model fitted to the data is presented. The slope of the line is the log-active cell fraction (solid lines: mean; dotted lines: 95% confidence interval; circles: values obtained in each cell dilution). A PML western blot from cells at the time of injection is presented in lower panel. (**d**,**e**) OSI formation in cell lines with high (BT549 and HBL100) and low (MCF7 and T47D) PML expression upon *PML* genetic silencing (MCF7 and T47D *n*=3, and BT549 and HBL100 *n*=6) (**d**) and 150 nM ATO (BT549 *n*=3, HBL100 *n*=5, MCF7 *n*=7 and T47D *n*=4) (**e**). A representative PML western blot out of three independent experiments is presented in lower panels. (**f**,**g**) Effect of 150 nM ATO on OSI formation (*n*=4) (**f**) and on PML levels (a representative western blot is presented out of four independent experiments) (**g**) in MCF7 parental cells and MCF7 metastatic sub-clone. Error bars represent s.e.m., *P* value (*P<0.05; ***P<0.001 compared with each control). Statistics test: one-tail unpaired *t*-test (**a**,**b**,**d**,**e**,**f**), *χ*^2^-test (**c**). ATO, arsenic trioxide; Met, metastatic; OSI, primary oncospheres; Par, parental; VC, vehicle control.

PML can be pharmacologically inhibited with arsenic trioxide (Trisenox, ATO), which induces SUMO-dependent ubiquitylation and proteasome-mediated degradation of the protein^38^,^39^. Similar to our results obtained by knocking down *PML* via shRNA, low doses of ATO decreased PML levels and exerted a negative effect on the OS formation capacity both in MDA-MB-231 and PDX44 cells (Fig. 4a,b). Moreover, ATO reduced the tumour formation capacity in a xenograft model derived from MDA-MB-231 cells in full coherence with the genetic approach (tumour-initiating cell frequency was estimated of 1/279 in vehicle and 1/703 in ATO; Fig. 4c; Supplementary Fig. 4a).

We hypothesized that cells with elevated PML would be ‘addicted'^40^ to the expression of the protein and hence be more sensitive to the action of PML inhibitors. To prove this notion, we studied additional cell lines with high (BT549, HBL100) or low (MCF7, T47D) PML expression. With this approach, we could demonstrate that the effect of *PML* silencing on the OS formation was exquisitely restricted to PML-high-expressing cells (Fig. 4d). This effect was recapitulated with ATO (Fig. 4e), where PML-low cells remained refractory to the drug in terms of the OS formation capacity.

Our results open a new avenue for the treatment of tumours that exhibit elevation in PML expression. *PML* elevation is predominant in ER-negative tumours (Supplementary Fig. 4b), which also present worse prognosis than ER-positive BCa^2^,^41^. Whereas luminal subtypes present better overall prognosis, there is a subset of patients within this subtype that exhibits aggressive disease^42^. We hypothesized that within this PML-low-expressing subtypes, the worse prognosis subgroup would exhibit increased PML levels. Indeed, MFS analysis within each intrinsic subtype confirmed that ER-positive BCa (luminal A and luminal B) contained a subset of patients with higher *PML* and worse prognosis (Supplementary Fig. 4c–g).

Our results in ER-positive tumours indicate that the PML expression is enriched in patients harbouring tumours of poor prognosis^2^,^3^. These results are coherent with our data in metastatic clone selection (Fig. 2c), suggesting that the acquisition of aggressive features is accompanied by the elevation of PML expression and ‘addiction' to the protein. We therefore sought to study whether metastatic ER-positive cell sub-clones, which present elevated PML expression, would exhibit sensitivity to PML inhibition, in contrast to the parental cells. Indeed, ATO reduced the OS formation selectively in PML-high-expressing metastatic cells derived from MCF7, whereas the parental cells remained refractory to the drug (Fig. 4f,g). Our results strongly suggest that PML elevation in BCa is associated to a dependence on its expression and hence enforces the need for patient stratification based on PML levels before the establishment of PML-directed therapies.

### PML regulates BCa-initiating cell function through SOX9

**Figure 5:**
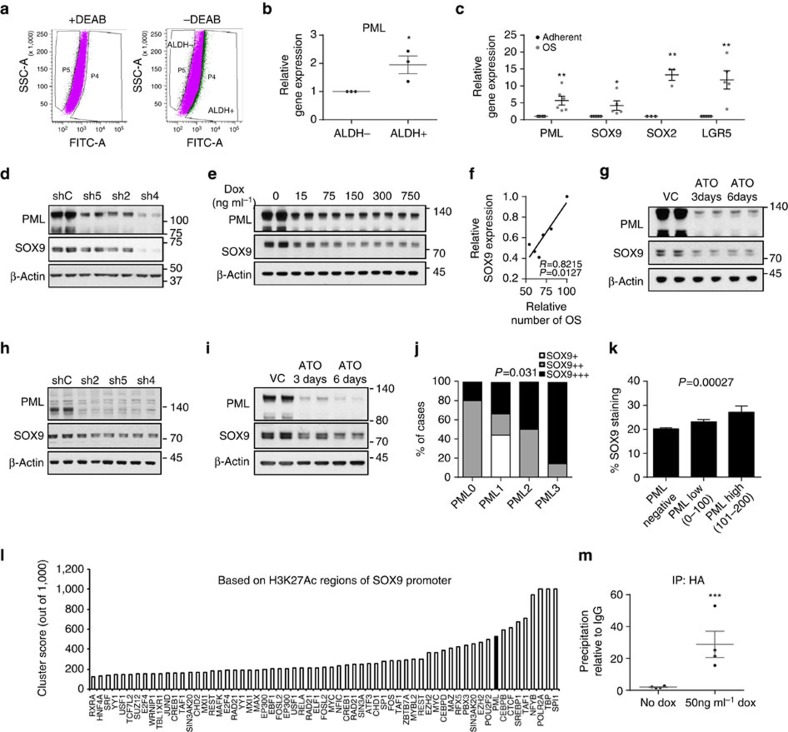
PML regulates SOX9 expression in breast cancer. (**a**) Flow cytometry analysis of MDA-MB-231 cells based on ALDH1 activity. (**b**) *PML* gene expression in the two populations sorted in a (*n*=3). (**c**) Expression of self-renewal-associated genes in OSI compared with adherent MDA-MB-231 cells (*PML n*=6, *SOX9* and *LGR5 n*=5 and *SOX2 n*=3). (**d**,**e**) Representative western blot out of four independent experiments depicting the downregulation of SOX9 protein upon constitutive (**d**) and inducible (**e**) *PML* silencing in MDA-MB-231 cells. (**f**) Correlation analysis of SOX9 protein densitometry from (**e**) and OSI formation in MDA-MB-231 cells (*n*=3). (**g**) Representative western blot out of four independent experiments depicting the downregulation of SOX9 protein upon 150 nM ATO treatment in MDA-MB-231 cells. (**h**,**i**) Representative western blot out of three independent experiments depicting the downregulation of SOX9 protein upon *PML* silencing (**h**) and 150 nM ATO treatment (**i**) in PDX44 cells. (**j**) PML and SOX9 immunoreactivity assed by immunohistochemistry in a panel of PDX samples (Table 1). (**k**) SOX9 immunoreactivity in patient biopsies with varying expression of PML in the Marseille cohort (*n*=737). (**l**) Cluster score of DNA-binding proteins in *SOX9* promoter region using ENCODE database. (**m**) SOX9 promoter region abundance in chromatin immunoprecipitation (ChIP) of exogenous HA-PMLIV using HA-tag antibody in MDA-MB-231 cells after induction with 50 ng ml^−1^ doxycycline for 3 days (*n*=4). Data were normalized to IgG (negative-binding control). Error bars represent s.e.m., *P* value (*P<0.05, **P<0.01, ***P<0.001 compared with control). Statistic test: one-tail unpaired *t-*test (**b**,**c**,**m**), Pearson correlation (**f**), *χ*^2^-test (**j**) and analysis of variance (**k**). ATO, arsenic trioxide; DEAB, diethylaminobenzaldehyde; dox, doxycycline; OSI, primary oncospheres; shC, Scramble shRNA; sh2, sh4 and sh5, shRNA against *PML*; VC, vehicle control.

To ascertain the molecular mechanism by which PML regulates BCa-initiating cell function, we first evaluated the expression levels of this gene in a sorted population of ALDH1-positive versus -negative MDA-MB-231 cells (Fig. 5a,b), and in adherent cultures versus OS (CIC-enriched cultures) (Fig. 5c). Strikingly, *PML* expression increased in both experimental approaches (Fig. 5b,c), together with the levels of well-established stem cell regulators (Fig. 5c). On this basis, we hypothesized that PML might control the expression of stem cell factors, as a mean to regulate BCa-initiating cell function. SOX9 is a recently described regulator of cell differentiation and self-renewal^9^,^10^,^11^ and is upregulated in BCa^12^,^13^,^14^. Constitutive (Fig. 5d; Supplementary Fig. 5a–b) and inducible (Fig. 5e) *PML* silencing exerted an inhibitory effect on SOX9 expression that correlated with the OS formation capacity (Fig. 5f). PML pharmacological inhibition also induced a decrease on SOX9 expression (Fig. 5g; Supplementary Fig. 5c–d). This regulatory activity was corroborated in the PDX44 cell line (Fig. 5h,i; Supplementary Fig. 5e), and in a correlative manner in the PDX data set (Fig. 5j), as well as in the aforementioned Marseille data set (Fig. 5k).

We next ascertained the molecular cues regulating *SOX9* expression downstream PML. Since the regulation was observed at the mRNA level, we interrogated *SOX9* promoter *in silico* and in public datasets. The ENCODE project has provided a vast amount of information about regulators and binding sites^43^. *SOX9* promoter exhibited a 2 kb region of acetylated H3K27 (H3K27Ac), which would indicate the proximal regulatory region. To our surprise, we found PML among the 10 proteins with highest confidence DNA-binding score in *SOX9* promoter region (Fig. 5l; cluster score=527 (refs 44, 45, 46)). There is limited evidence of the capacity of PML to regulate gene expression in concordance with transcription factors through association with DNA^47^,^48^,^49^. We performed chromatin immunoprecipitation (ChIP) analysis of ectopically expressed and endogenous PML, as well as SOX9 expression analysis in these conditions. We confirmed that PML is in close proximity to *SOX9* promoter region and that its ectopic expression upregulates *SOX9* transcript and protein levels (Fig. 5m; Supplementary Fig. 5f–j). It is worth noting that PML does not present a canonical DNA-binding domain, and it is therefore plausible that it lies in close proximity to *SOX9* promoter through the interaction with intermediary DNA-binding proteins.

**Figure 6:**
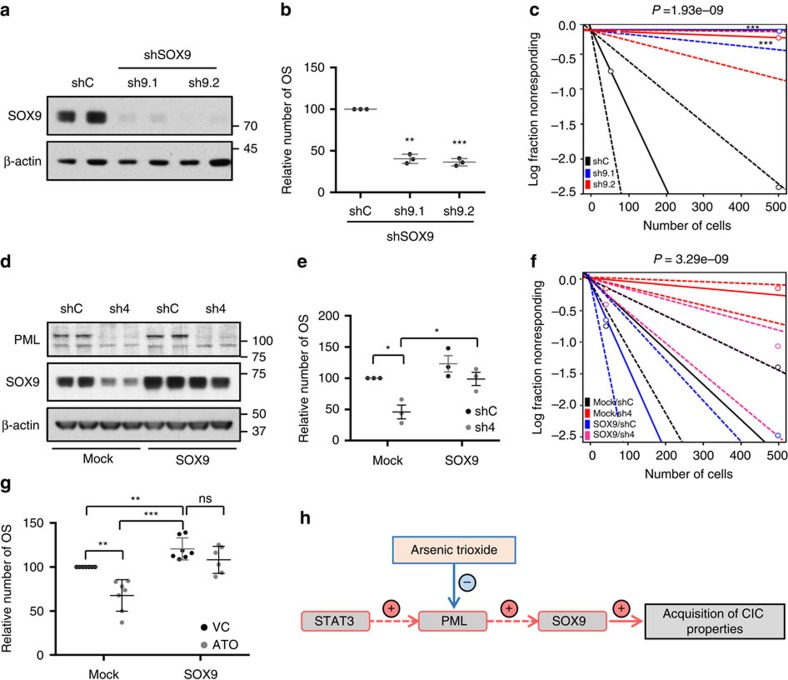
SOX9 is critical for the regulation of breast cancer-initiating capacity downstream PML. (**a**,**b**) Effect of *SOX9* silencing with two shRNA (sh9.1 and sh9.2) on SOX9 protein expression (representative western blot out of three independent experiments) (**a**) and on OSI formation (*n*=3) (**b**) in MDA-MB-231 cells. (**c**) Limiting dilution experiment after xenotransplantation. Nude mice were inoculated with 500,000 or 50,000 MDA-MB-231 cells (*n*=8 injections per experimental condition). Tumour-initiating cell number was calculated using the ELDA platform. A log-fraction plot of the limiting dilution model fitted to the data is presented. The slope of the line is the log-active cell fraction (solid lines: mean; dotted lines: 95% confidence interval; circles: values obtained in each cell dilution). (**d**,**e**) Effect of ectopic SOX9 expression on the consequences of *PML* silencing. Representative western blot (out of three independent experiments) depicting expression of PML and SOX9 (endogenous and ectopic protein are detected) (**d**) and OSI formation (*n*=3) (**e**) in the different experimental conditions in MDA-MB-231 cells. (**f**) Limiting dilution experiment to assess frequency of tumour-initiating cells after xenotransplantation. Nude mice were inoculated either with 500,000 or 50,000 MDA-MB-231 cells (*n*=12 per experimental condition, except in shC/Mock and sh4/SOX9, *n*=16). Tumour-initiating cell number was calculated using the ELDA platform as in **c**. (**g**) OSI formation in MDA-MB-231 cells transduced with the indicated constructs (mock, *SOX9*) and treated with vehicle or 150 nM ATO (*n*=6). (**h**) Diagram of the molecular mechanism by which PML controls the expression of the stem cell factor SOX9 to regulate BCa-initiating cell function. Error bars represent s.e.m., *P* value (*P<0.05; **P<0.01; ***P<0.001 compared with its control or as indicated). Statistic test: one-tail unpaired *t-*test (**b**,**e**,**g**) and *χ*^2^-test (**c**,**f**). ATO, arsenic trioxide; OSI, primary oncospheres; shC, Scramble shRNA; sh9.1 and sh9.2, shRNA against *SOX9*; sh4, shRNA against *PML*; VC, vehicle control.

The regulation of *SOX9* led us to hypothesize that this transcription factor mediated the effects of PML on the regulation of CIC function. On the one hand, we ascertained whether *SOX9* silencing would recapitulate the effects of PML inhibition. We set up two shRNAs targeting *SOX9* (Fig. 6a) that exhibited a potent effect on primary (Fig. 6b) and secondary (Supplementary Fig. 6a) OS formation. Moreover, *SOX9* silencing in MDA-MB-231 cells reduced the tumour formation capacity *in vivo* (tumour-initiating cell frequency was estimated of 1/71.7 in shC, completely abolished (1/infinite) in sh9.1 and 1/4145.5 in sh9.2; Fig. 6c; Supplementary Fig. 6b–c), in agreement with other reports^12^.

On the other hand, we evaluated the capacity of ectopically expressed SOX9 to bypass the effects of PML silencing on CIC function. Ectopic SOX9-expressing BCa cells were refractory to *PML* genetic inhibition in terms of the OS formation (Fig. 6d,e) and tumour formation (tumour-initiating cell frequency was estimated of 1/139.8 in shC/Mock, 1/57.5 in shC/SOX9, 1/1506 in sh4/Mock and 1/270.8 in sh4/SOX9; Fig. 6f; Supplementary Fig. 6d). Importantly, the *in vitro* observation was recapitulated in ATO-treated cells (Fig. 6g).

These data reveal a novel molecular mechanism by which PML controls the expression of the stem cell factor SOX9 to regulate BCa-initiating cell function (Fig. 6h). It is worth noting that we found PML at the promoter region of other stem cell genes, such as *LGR5* (Supplementary Fig. 6e–g), indicating that the capacity of this protein to regulate CIC function could involve a larger and more complex transcriptional program.

## Discussion

Finding successful targeted treatment strategies for women at risk of metastatic BCa is of outstanding clinical interest. Our data unveil the therapeutic potential of targeting PML in combination with a stratification companion that identifies patients harbouring PML-high-expressing BCa.

We demonstrate that PML targeting impacts on BCa-initiating cell function, and hence on cancer initiation and dissemination. In addition, we observed that PML expression is increased in BCa-initiating cells, highly metastatic sub-clones and in BCa patients at risk of metastasis. These data suggest that, in a subset of BCas, PML sustains the function of BCa-initiating cells and in turn supports the metastatic dissemination capacity^6^.

We show that PML-directed therapies are efficient in BCa cells with elevated expression of the protein. Such phenomenon has been defined as ‘addiction'^40^,^50^,^51^, and it represents an exciting avenue in the establishment of novel therapeutic initiatives. Importantly, targeted therapies have been particularly successful when combined with a predictive biomarker. The availability of a clinically validated protocol to detect PML immunoreactivity^52^ offers a unique opportunity to define the patients that would benefit from therapies based on PML inhibition. In addition, our proof-of-concept demonstration of the therapeutic efficacy of PML pharmacological inhibition with ATO indicates that (1) repositioning of ATO (that is currently used in the treatment of acute PML) for BCa therapy is a viable approach, (2) there is strong support for the development of novel and more effective PML inhibitors and (3) the identification of combined therapies with PML inhibitors in BCa is a novel and exciting area of investigation.

Mechanistically, our data demonstrate that PML is in close proximity to the promoter region of *SOX9*, and positively regulates the expression of the gene. SOX9 has been recently established as a central regulator of normal and cancer stem cells^9^,^10^,^11^,^12^,^13^,^53^,^54^,^55^,^56^,^57^,^58^,^59^,^60^,^61^. This activity is executed in part through the functional interplay with epithelial-to-mesenchymal transition regulators such as SLUG^12^,^53^,^56^. In BCa, SOX9 is found overexpressed in the TNBC subtype, and regulates the WNT/beta-catenin pathway^14^. In addition, this transcription factor is a main driver of the transcriptional signature of this subtype of BCa^62^. All these features make SOX9 an ideal target for BCa therapy. However, development of small molecules targeting transcription factors has been an outstanding challenge with limited success^63^. Our data demonstrating that PML sustains SOX9 expression in aggressive BCa opens the possibility to bypass this limitation and inhibit the function of the transcription factor through upstream PML targeting.

In summary, our data provide proof-of-concept demonstration of the fact that PML-inhibiting compounds could exhibit strong potential for BCa therapy upon PML-based stratification.

## Methods

### Cell culture

MDA-MB-231, BT594, HBL100, MCF7 and T47D cell lines were obtained from the American Type Culture Collection (Manassas, VA, USA) or from Leibniz-Institut—Deutsche Sammlung von Mikroorganismen und Zellkulturen GmbH (DMSZ, Germany), who provided an authentication certificate. None of the cell lines used in this study was found in the database of commonly misidentified cell lines maintained by ICLAC and NCBI biosample. PDX44-derived cell line was generated by Dr Ibrahim and Dr Serra starting from xenograft tumours. Cell lines were routinely monitored for mycoplasma contamination and quarantined, while treated if positive. All cell lines were maintained in DMEM media supplemented with 10% (v/v) foetal bovine serum and 1% (v/v) penicillin–streptomycin. OS formation assays were carried out as previously described^64^. In brief, single-cell suspensions were plated in six-well tissue culture plates covered with poly-2-hydroxyethyl-methacrylate (Sigma, St Louis, MO) to prevent cell attachment, at a density of 3,000 cells per ml in serum-free DMEM supplemented with 1% penicillin/streptomycin, 1% B27 (Invitrogen, Carlsbad, CA, USA), 10 ng ml^−1^ epidermal growth factor (EGF) (Sigma, St. Louis, MO) and 2 ng ml^−1^ fibroblast Growth Factor, basic (FGFb) (Invitrogen, Carlsbad, CA, USA). After 6 days in culture, OS were counted using a light microscope. For secondary OS formation, following the same protocol, 100,000 cells were plated in 100 mm dishes and collected by gentle centrifugation (200*g*) and dissociated enzymatically (5 min in 1:1 TrypLE solution at 37 °C, Life Technologies, cat: 12604013) and single cells were re-plated at a density of 3,000 cells per ml in six-well tissue culture plates for 6 days.

### Generation of stable cell lines

293FT cells were used for lentiviral production. Lentiviral vectors expressing shRNAs against human *PML*, *STAT3* and *SOX9* from the Mission shRNA Library were purchased from Sigma-Aldrich or Addgene. Cells were transfected with lentiviral vectors following standard procedures, and viral supernatant was used to infect cells. Selection was done using puromycin (2 μg ml^−1^) for 48 h. As a control, a lentivirus with scrambled shRNA (shC) was used. Short hairpins sequence: sh1PML (TRCN0000003865): CCGGCAATACAACGACAGCCCAGAACTCGAGTTCTGGGCTGTCGTTGTATTGTTTTT, sh2PML (TRCN0000003865): CCGGCAATACAACGACAGCCCAGAACTCGAGTTCTGGGCTGTCGTTGTATTGTTTTT; sh4PML (TRCN 0000003867): CCGGGCCAGTGTACGCCTTCTCCATCTCGAGATGGAGAAGGCGTACACTGGCTTTTT; sh5PML (TRCN 0000003867): CCGGGTGTACCGGCAGATTGTGGATCTCGAGATCCACAATCTGCCGGTACACTTTTT; shC: CCGGCAACAAGATGAAGAGCACCAACTCGAGTTGGTGCTCTTCATCTTGTTG. sh41STAT3 (TRCN0000020841): CCGGGCTGAAATCATCATGGGCTATCTCGAGATAGCCCATGATGATTTCAGCTTTTT; sh43STAT3 (TRCN0000020843): CCGGGCAAAGAATCACATGCCACTTCTCGAGAAGTGGCATGTGATTCTTTGCTTTTT. sh1SOX9 (Addgene, GenBank ID: RHS3979-9587792; GCATCCTTCAATTTCTGTATA); sh2SOX9 (TRCN0000342824): CCGGCTCCACCTTCACCTACATGAACTCGAGTTCATGTAGGTGAAGGTGGAGTTTTTG. Sub-cloning of shC and sh4PML into pLKO-Tet-On vector was done introducing AgeI and EcoRI in the 5′-end of top and bottom shRNA oligos, respectively (following the strategy provided by Dr Dmitri Wiederschain^65^, Addgene plasmid: 21915). HA-PMLIV was sub-cloned into a TRIPZ vector using Age1–Mlu1 sites.

### Immunoassays

Western blot analysis was carried out as previously described^20^. Uncropped scans are provided as part of the Supplementary Information (Supplementary Fig. 7). In brief, cells were seeded on six-well plates and 4 days (unless otherwise specified) after seeding cell lysates were prepared with RIPA buffer (50 mM TrisHCl pH 7.5, 150 mM NaCl, 1 mM EDTA, 0.1% SDS, 1% Nonidet P40, 1% sodium deoxycholate, 1 mM sodium fluoride, 1 mM sodium orthovanadate, 1 mM beta-glycerophosphate and protease inhibitor cocktail; Roche). The following antibodies were used for western blotting: rabbit polyclonal anti-PML, 1:1,000 dilution (cat: A301-167A; Bethyl laboratories), rabbit polyclonal anti-phospho-STAT3 (Tyr705) 1:1,000 dilution and total STAT3 1:1,000 dilution (cat: 9145, 9132 respectively; Cell Signaling), rabbit polyclonal anti-SOX9 1:2,000 dilution (cat: AB5535; CHEMICON International), HA-Tag polyclonal antibody 1:2,000 dilution (cat: C29F4, Cell Signaling Technology, Inc) and mouse monoclonal anti-beta-ACTIN 1:2,000 dilution (clone: AC-74, catalogue: A5316, Sigma-Aldrich). After standard SDS–polyacrylamide gel electrophoresis and western blotting techniques, proteins were visualized using the enhanced chemiluminescence (ECL) system.

For immunofluorescence, cells were seeded on glass cover slips in 24-well plates and 4 days after seeding, cells were fixed with 4% paraformaldehyde (15 min), PBS (three times wash), 1% Triton X-100 (5 min), PBS (three times wash), 10% goat serum (1 h) and anti-PML antibody 1:100 ditution (catalogue A301-167A; Bethyl laboratories) was added overnight (4°C) in goat serum. Cover slips were washed with PBS three times and incubated with secondary antibody (anti-rabbit Alexa488; Invitrogen-Molecular Probes) for 1 h (room temperature). Cover slips were washed with PBS three times, and 4,6-diamidino-2-phenylindole added to stain nuclei (10 min), followed by mounting with Mowiol. Immunofluorescence images were obtained with an AxioImager D1 microscope.

For immunohistochemistry, tissues were fixed in 10% neutral-buffered formalin and embedded in paraffin according to standard procedures. Three to four μm-thick sections were stained for PML (clone PG-M3 Santa Cruz Biotechnology Inc, sc-966, 1:200 dilution), and vimentin (1:1,000, NCL-L-VIM-V9, Novocastra). Antigen retrieval was performed with citrate buffer (pH 6). Detection was performed with the ABC Kit from Vector Laboratories and 3,3′-diaminobenzidine (DAB)-based development. Sections were counterstained with haematoxylin. The PML general immunoreactivity scoring system (used in Fig. 1h) is described in ref. 20. For the Marseille data set, PML (1:200), SOX9 (1:400, Millipore) and phospho-STAT3 (Tyr705) (1:100, Cell Signalling: M9C6) immunostaining was performed as reported^11^,^20^. The percentage of PML-high (Ph) and -low (Pl) immunoreactive tumour cells in the Marseille data set was quantified separately and the *h*-score was calculated, attributing a relative value of 1 × to Pl and 2 × to Ph intensity nuclear signal (*h*=(1 × Pl)+(2 × Ph)). For SOX9 automated quantification and construction of tissue microarrays (TMAs) in the Marseille data set was carried out as reported^23^. In brief, cores were punched from the selected paraffin blocks, and distributed in new blocks including two cores of 0.6 mm diameter for each tumour. All the TMA blocks were stored at 4 °C. TMA serial tissue sections were prepared 24 h before immunohistochemistry processing and stored at 4 °C. The immunoperoxidase procedures were performed using an automated Ventana Benchmark XT auto-stainer. This device allowed identical well-controlled procedures for antigen retrieval and Ventana kits.

### Quantitative real-time PCR

Cells were seeded as for western blot. Total RNA was extracted from cells using NucleoSpin RNA isolation kit from Macherey-Nagel (ref: 740955.240C). Complementary DNA was produced from 1 μg of RNA using qScript cDNA SuperMixt (Quanta Bioscience, ref: 95048). Taqman probes were obtained from Applied Biosystems. Amplifications were run in a Viia7 Real-Time PCR System (Applied Biosystems) using the following probes: *PML* (Hs00971694_m1, cat: 4331182) and *SOX9* (Hs01001343_g1, cat: 4331182). For *STAT3*, *SOX2* and *LGR5* amplification, Universal Probe Library (Roche) primers and probes were employed (*STAT3*, For: cccttggattgagagtcaaga, Rev: aagcggctatactgctggtc; probe: 14; *SOX2*, For: gggggaatggaccttgtatag, Rev: gcaaagctcctaccgtacca; probe: 65; *LGR5*, For: accagactatgcctttggaaac, Rev: ttcccagggagtggattctat; probe: 78). *β-actin* (Hs99999903_m1, cat: 4331182) and *GAPDH* (Hs02758991_g1, cat: 4331182) housekeeping assays from Applied Biosystems showed similar results (all quantitative PCR with reverse transcription data presented were normalized using *GAPDH*).

### ALDH1 activity by FACS

To measure the ALDH1 activity present in the cells, the ALDEFLUOR assay was carried out according to manufacturer's (Stemcell Technologies) guidelines. In brief, dissociated MDA-MB-231 cells were resuspended in ALDEFLUOR assay buffer at a final concentration of 1.10^6^ ml^−1^. ALDH substrate, bodipyaminoacetaldehyde was added to the cells at a final concentration of 1.5 mM. Immediately, half of the cells were transferred to an Eppendorf tube containing a two fold molar excess of the ALDH inhibitor, diethylaminobenzaldehyde. Both tubes were incubated for 45 min at 37 °C, and after this incubation cells were centrifuged at 250*g* for 5 min at 4 °C and resuspended in ice-cold ALDEFLUOR assay buffer. Cells were analysed using a FACSAria1 (Becton Dickinson) flow cytometer. DRAQ7 (BiostatuS) was added prior analysis to each tube for dead cell exclusion. FACSAria1 was also used for sorting cells. Data were analysed using the FACSDiva software.

### Reagents

For *in vitro* experiments, SI3-201 (Sigma-Aldrich, SML0330) was prepared at 10 mg ml^−1^ in dimethylsulfoxide and used at the indicated concentrations. TG101348 (Santa Cruz, sc-364740) was prepared 100 mg ml^−1^ in dimethylsulfoxide and used at the indicated concentrations. ATO (Sigma-Aldrich) was prepared at a concentration of 100 mM in NaOH 1 N and subsequently diluted to 0.1 mM in PBS for a 1,000 × working solution. ATO was used at 150 nM either 3 or 6 days as indicated in figure legends. For *in vivo* experiments a dose of 5 mg kg^−1^ per day was intraperitoneally administered.

### Mice

Xenograft experiments were carried out following the ethical guidelines established by the Biosafety and Welfare Committee at CIC bioGUNE and Biodonostia Institute. The procedures employed were carried out following the recommendations from AAALAC. Xenograft experiments were performed as previously described^66^, injecting either 5.10^5^ or 5.10^4^ cells per condition (unless otherwise specified), four injections per mouse. Metastasis experiment was approved by the institutional animal care and use committee of IRB-Barcelona. For tail vein injections, cells were resuspended in PBS and injected into tail vein of mice using a 26 G needle (1.2 × 10^5^ cells per mouse), as previously described^25^. Cell lung colonization capacity was scored 21 days post inoculation by human vimentin. PML expression was scored as undetectable (PML 0) and detectable (PML 1+, 2+ and 3+). All mice (female Hsd:Athymic Nude-Foxn1 nu/nu) were inoculated at 8–12 weeks of age.

### ChIP

ChIP was performed using the SimpleChIP Enzymatic Chromatin IP Kit (cat: 9003, Cell Signaling Technology, Inc). MDA-MB-231 cells were grown in 150 mm dishes either with or without 50 ng ml^−1^ doxycycline during 3 days. Cells from three 150 mm dishes (2.5 × 10^7^ cells) were cross-linked with 35% formaldehyde for 10 min at room temperature. Glycine was added to dishes, and cells incubated for 5 min at room temperature. Cells were then washed twice with ice-cold PBS, and scraped into PBS+PMSF. Pelleted cells were lysed and nuclei were collected following manufacturer's instructions. Nuclear lysates were digested with micrococcal nuclease for 20 min at 37 °C and then sonicated in 500 μl aliquots on ice for three pulses of 15 s using a Branson sonicator. Cells were held on ice for at least 1 min between sonications. Lysates were clarified at 11,000*g* for 10 min at 4 °C, and chromatin was stored at −80 °C. HA-Tag polyclonal antibody (cat: C29F4, Cell Signaling Technology), rabbit polyclonal anti-PML (cat: A301-167A; Bethyl laboratories) and IgG antibody (cat: 2729, Cell Signaling Technology, Inc), were incubated overnight (4 °C) with rotation and protein G magnetic beads were incubated 2 h (4 °C). Washes and elution of chromatin were performed following manufacturer's instructions. DNA quantification was carried out using a Viia7 Real-Time PCR System (Applied Biosystems) with SybrGreen reagents and primers that amplify the predicted PML binding region to *SOX9* promoter (chr17:70117013-70117409) as follows: left primer: ccggaaacttttctttgcag and right primer: cggcgagcacttaggaag.

### Patient data sets, bioinformatics and statistical analysis

All studies involving human subjects were approved by the corresponding committees with informed consent as stated in the original publications^3^,^23^,^25^. The use of MSK/EMC and Marseille cohorts were previously described^23^,^25^. For MSK/EMC, MFS curves were plotted using Kaplan–Meier estimates and compared using the Gehan–Breslow–Wilcoxon test. Two groups were compared using mean *PML* expression values as the cutoff between PML high and low. Kaplan–Meier survival and correlation analysis in patient samples: publicly available and clinically annotated BCa cohorts with gene expression profiles (GSE2603, GSE2034, GSE5327 and GSE12276) were pooled as described above. To remove systematic biases, before merging the expression measurements were converted to *z* scores for all genes. For intrinsic subtype classification, we carried out the following analysis: for luminal genes, *ESR1* and *PGR1* presented a bimodal distribution. We used package mclust to fit a mixture of normal distributions with two components and obtain the posterior probability that each patient belongs to the luminal low and luminal high components. A patient was considered luminal low if the posterior probability of belonging to this group was >80%. The same criterion was used for luminal high. When a patient was neither luminal high nor luminal low, it was considered luminal intermediate. Proliferation status (Prol) and *ERBB2* expression did not present a bimodal distribution. Therefore, half of the patients with lowest mean values were considered proliferation low. The rest were considered proliferation high. After defining high and low populations for each parameter, the subtypes were constructed as follows: luminal A: Prol low, *ESR1* intermediate or high, luminal intermediate or high; luminal B: Prol high, *ESR1* intermediate or high, luminal intermediate or high; *HER2* enriched: Prol high, *ESR1* intermediate or low, luminal intermediate or low; *ERBB2* high; basal like: Prol high, *ESR1* low, luminal low; *ERBB2* low, *PGR1* low. Sixty-four patients could not be assigned to any subtype according to PAM50's classification. A Cox proportional hazards model was fitted to compute HR. Likelihood ratio tests were performed to compute *P* values. The HR was checked for constancy over time, fulfilling Cox model assumptions.

For Curtis data set patients, RNA was extracted from 1,980 tumours as described^3^. RNA hybridizations were performed using Illumina HT-12 v3 platform and analysed using the bioconductor bead array package^67^. The BASH algorithm^68^ was applied to correct for spatial artefacts in the arrays. Bead-level data were summarized and re-annotated as described in ref. 3. Log-intensity values for *PML* expression were scaled to *z* scores. Probe selection was performed on the basis of probe quality, 3′-position, no other genomic matches and no single-nucleotide polymorphisms in the region. On the basis of these criteria, PML probe ILMN_1731299 was selected for analysis. Survival analysis was done using as endpoints MFS at 5 years (distant metastasis as event). Two groups were compared using mean *PML* expression values as the cutoff between PML high and low. We used the log-rank test as implemented in the survival R package^69^.

For therapy response analysis, publicly available data sets (GSE22093 and GSE23988) were downloaded from Gene Expression Omnibus (GEO), and subjected to background correction, log_2_ transformation and quartile normalization.

For correlation analysis with *STAT3* signatures, gene sets were extracted (ref. 37, and http://software.broadinstitute.org/gsea/msigdb/cards/V$STAT3_01) and average signal value in the MSK/EMC data set was calculated. These values were used to perform the correlation analysis with PML signal values (Pearson correlation).

No statistics were applied to determine sample size. The experiments were not randomized. The investigators were not blinded to allocation during experiments and outcome assessment. Data analysed by parametric tests are represented by the mean±s.e.m. of pooled experiments unless otherwise stated. *n* values represent the number of independent experiments performed or the number of individual mice or patient specimens. For each independent *in vitro* experiment, at least three technical replicates were used and a minimum number of three experiments were performed to ensure adequate statistical power. Analysis of variance test was used for multi-component comparisons and Student's *t*-test for two-component comparisons. In the *in vitro* experiments, normal distribution was confirmed or assumed (for *n*<5) and Student's *t*-test was applied for two-component comparisons. Two-tailed statistical analysis was applied for experimental design without predicted result, and one tail for validation or hypothesis-driven experiments. The confidence level used for all the statistical analyses was of 0.95 (alpha value=0.05). Tumour-initiating cell frequency was estimated using ELDA software as previously described^70^.

### Data availability

Data from public repositories analysed throughout this manuscript (see the ‘Patient data sets, bioinformatics and statistical analysis' section) is available as indicated in the referenced publications.

## Additional information

**How to cite this article**: Martín-Martín, N. *et al*. Stratification and therapeutic potential of PML in metastatic breast cancer. *Nat. Commun.* 7:12595 doi: 10.1038/ncomms12595 (2016).

## Supplementary Material

Supplementary Information Supplementary Figures 1-7.

## Figures and Tables

**Table 1:** PDX characterization based on BCa subtype (intrinsic subtype is presented in brackets).

**PDX**	**Subtype**	**PML**
31	TNBC (HER2 enriched)	−
102	ER+ (basal like)	−
131	ER+ (luminal B)	−
156	ER+ (basal like)	−
197	TNBC (basal like)	−
4	ER+ (luminal B)	+
6	ER+ (luminal A)	+
10	HER2+ (HER2 enriched)	+
39	ER+ (luminal B)	+
60	ER+ (basal like)	+
98	ER+ (basal like)	+
136	TNBC (basal like)	+
137	TNBC (basal like)	+
161	ER+ (luminal B)	+
93	TNBC (NA)	++
179	TNBC (NA)	++
44	TNBC (basal like)	+++
88	TNBC (basal like)	+++
89	TNBC (NA)	+++
94	TNBC (basal like)	+++
124	TNBC (basal like)	+++
127	TNBC (basal like)	+++
167	TNBC (basal like)	+++

ER, oestrogen receptor; NA, not applicable; TNBC, triple-negative breast cancer.
