# Supplementary material for: Stratification and therapeutic potential of PML in metastatic breast cancer
Source: Nat Commun. 2016 Aug 24;7:12595. doi: 10.1038/ncomms12595 (PMC4999521; doi:10.1038/ncomms12595)
Supplement: Supplementary Information — Supplementary Figures 1-7. [file ncomms12595-s1.pdf]

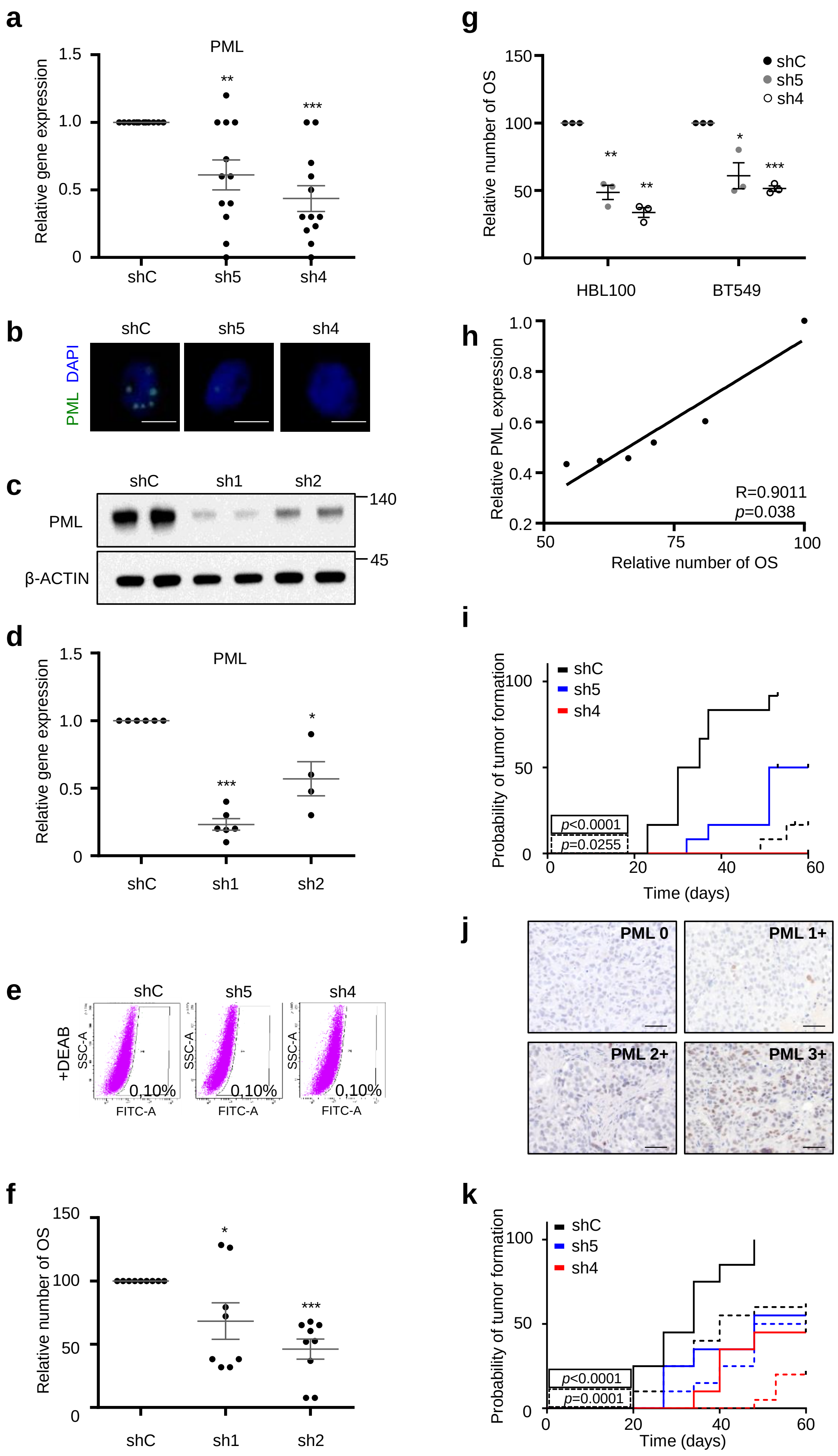

Supplementary Figure 1. Extended data related to Figure 1.

(a) *PML* gene expression upon shRNA-mediated silencing with sh5 and sh4 in MDA-MB-231 cells (n=12). **(b)** Representative magnified images out of three independent experiments of PML immunofluorescent reactivity upon shRNA-mediated silencing in MDA-MB-231 cells. **(c)** Representative Western blot out of four independent experiments showing PML protein expression upon silencing with two different short hairpins (sh1 and sh2) in MDA-MB-231 cells. Scale bar: 10μm. **(d)** *PML* gene expression upon silencing with sh1 and sh2 in MDA-MB-231 cells (sh1 n=6, sh2 n=4). **(e)** DEAB treatment in MDA-MB-231 cells stained for Aldefluor substrate as a gating control for ALDH1 activity in Fig. 1b-c (n=3). **(f)** Effect of *PML* silencing with sh1 and sh2 on OSI formation in MDA-MB-231 cells (sh1 n=8, sh2 n=9). **(g)** OSI formation in the indicated cell lines upon *PML* silencing (n=3). **(h)** Correlation analysis of PML protein densitometry from Fig. 1e and OSI formation in MDA-MB-231 cells (n=3). **(i)** Kaplan-Meier curves of tumour formation after inoculation with 500.000 (solid line) or 50.000 (dashed line) MDA-MB-231 cells transduced with the indicated shRNAs (n=12 injections per experimental condition). **(j)** PML expression assed by IHC in PDX model: undetectable (PML 0) and differing abundance (PML 1+, 2+, 3+). Representative immunoreactivity images are shown in lower panels. Scale bar: 50μm. **(k)** Kaplan-Meier curves of tumour formation after inoculation with 100.000 (solid line) or 10.000 (dashed line) PDX44 cells transduced with the indicated shRNAs (n=20 injections per experimental condition). Error bars represent s.e.m. p, p-value (*p< 0.05, **p< 0.01, ***p< 0.001 compared to each control). Statistics test: one tail unpaired T test (a, d, f, g), Pearson´s correlation (h) and Log-rank (Mantel-Cox) test (i, j). shC: Scramble shRNA, sh1, sh2, sh4 and sh5: shRNA against *PML*, OSI: primary oncospheres, DEAB: diethylaminobenzaldehyde.

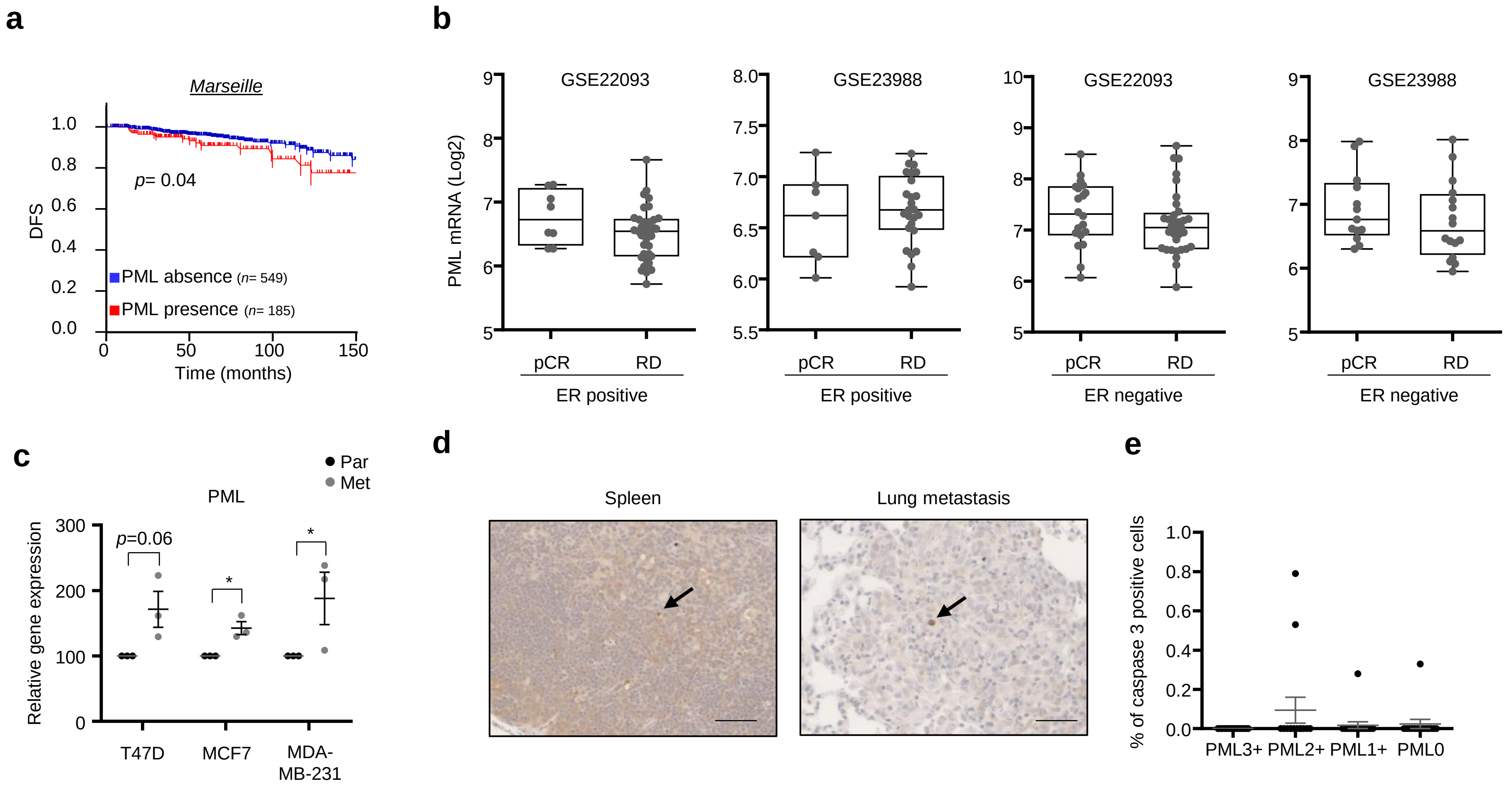

Supplementary Figure 2. Extended data related to Figure 2.
(a) Kaplan-Meier representations of DFS based on PML protein (PML absence vs. PML presence) expression in Marseille dataset (n=734). (b) Box plots of PML expression in pCR (pathologic complete response) and in RD (residual disease) upon treatment regime in two independent data sets (GSE22093 and GSE23988). (c) PML gene expression in cell line sub-clones selected for high metastatic potential (n=3). (d) Representative immunohistochemistry images of spleen and lung metastatic foci of tail vein-injected mice (Fig. 2d-f) showing cleaved caspase-3 expression (arrow). (e) Percentage of cleaved caspase-3 positive cells among the metastatic foci with different PML immunoreactivity. Scale bar: 50μm. Error bars represent s.e.m. p, p-value (*p< 0.05, compared with each control). Statistical test: Gehan-Breslow-Wilcoxon test (a), two tail unpaired T test (b) and one tail unpaired T test (c). DFS: disease free survival, Par: parental, Met: metastatic, ER: oestrogen receptor.

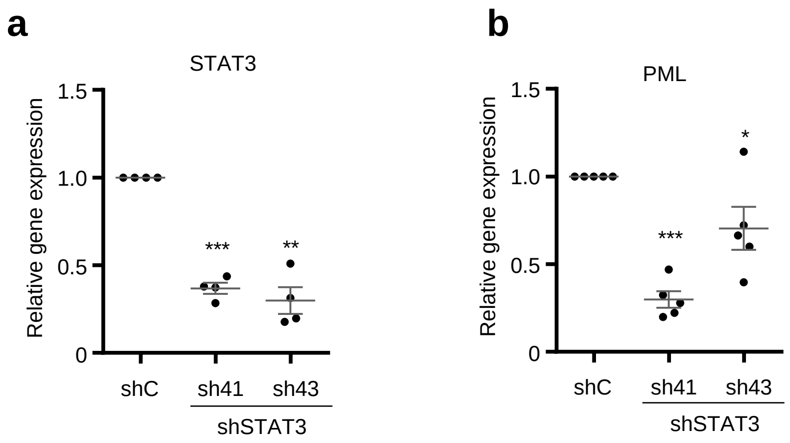

Supplementary Figure 3. Extended data related to Figure 3.
(a) *STAT3* gene expression upon silencing with sh41 and sh43 in MDA-MB-231 cells (n=4). **(b)** *PML* gene expression upon *STAT3* silencing with sh41 and sh43 in MDA-MB-231 cells (n=5). Error bars represent s.e.m. p, p-value (*p< 0.05, **p< 0.01, ***p< 0.001 compared with each control). Statistics test: one tail unpaired T test. shC: Scramble shRNA, sh41 and sh43: shRNA against *STAT3*.

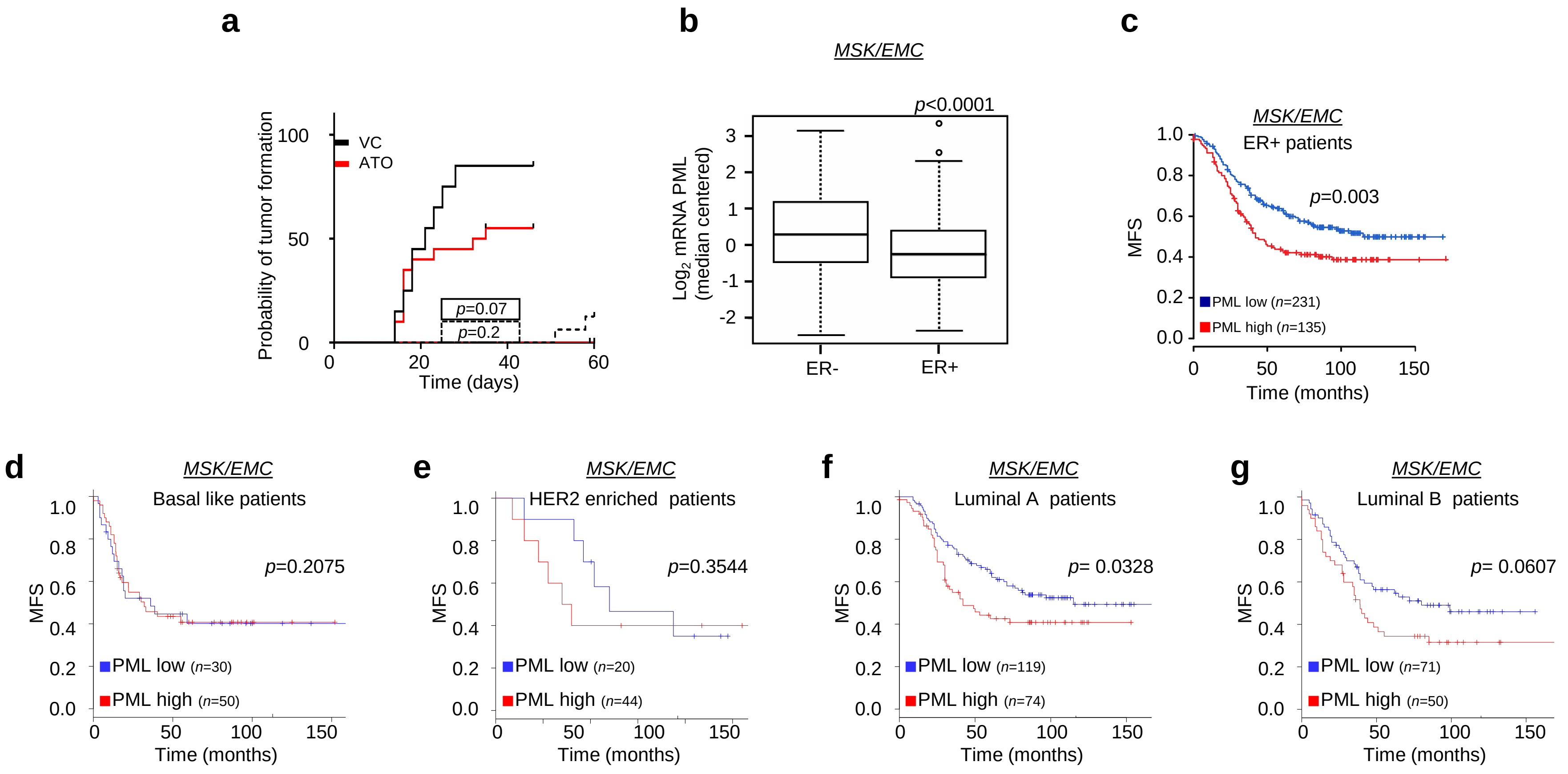

Supplementary Figure 4. Extended data related to Figure 4.
(a) Kaplan-Meier curves of tumour formation after inoculation with 500.000 (solid line) or 50.000 (dashed line) MDA-MB-231 cells treated as indicated (n=20 injections per experimental condition. **(b)** Box-plot showing *PML* expression in ER- and ER+ patient specimens from MSK/EMC dataset. **(c-g)** Kaplan-Meier MFS representations based on *PML* RNA (PML high: above the mean expression; PML low: below the mean expression) in ER+ **(c)**, Basal-like **(d)**, HER2-enriched **(e)**, Luminal A **(f)** and Luminal B **(g)** subtypes. p, p-value. Statistics test: Log-rank (Mantel-Cox) test (a), two tail unpaired T test (b) and Gehan-Breslow-Wilcoxon test (c-g). VC: vehicle control, ATO: arsenic trioxide, MFS: metastasis-free survival, ER: oestrogen receptor.

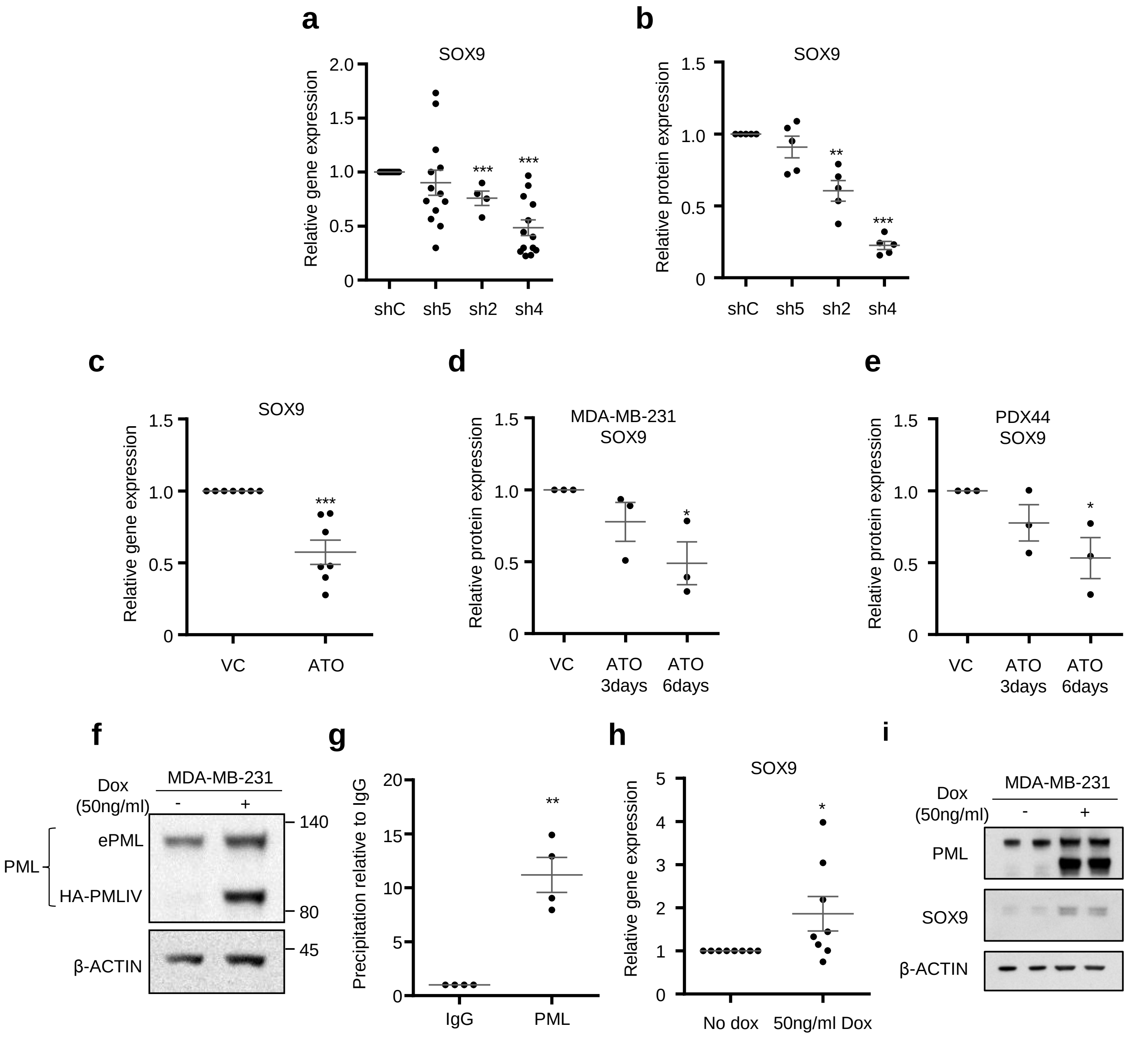

j

5´ GTCACCCAGCCCAGTGCCACAATCCTCCTCCCTCCCCAAAATCGGGTCCAATCAGCTGCCTGCCAACCCTGGGACTGCTGTGC
TGTGATTGGCGGGTGGCTCTAAGGTGAGGCGGAGTATTTATTAAAGAGACCCTGGGCTGGGAGTTGGAGAGCCGAAAGCGGAGC
TCGAAACTGACTGGAAACTTCAGTGGCGCGGAGACTCGCCAGTTTCAACC<u>*CCGGAAACTTTTCTTTG*</u>CAGGAGGAGAAGAGAAGG
GGTGCAAGCGCCCCCA<u>**CTTTTGCTCTTTTTCCTCCCCTCCTCCTCCTCTCCAATTCGCCTCCCCCCACTTGGAGCGGGCAGCTGTG**</u>
<u>**AACTGGCCACCCCGCGCCTTCCTAAGTGCTCGCC**</u>GCGGTAGCCGGCCGACGCGCCAGCT **3´**

Supplementary Figure 5. Extended data related to Figure 5.
(a) *SOX9* gene expression upon *PML* silencing with 3 different shRNAs in MDA-MB-231 cells (sh2 n=4, sh4 and sh5 n=13). **(b)** SOX9 protein densitometry in MDA-MB-231 upon *PML* silencing (n=5). **(c)** *SOX9* expression upon 150 nM ATO treatment for 6 days in MDA-MB-231 cells (n=7). **(d-e)** SOX9 protein densitometry in MDA-MB-231 **(d)** and PDX44 **(e)** cell lines after 150 nM ATO treatment during 3 or 6 days as indicated (n=3). **(f)** Representative Western blot out of four independent experiments of endogenous PML (ePML) and HA-PMLIV protein expression after 72 h of doxycycline treatment (50 ng/ml) in MDA-MB-231 cells. **(g)** *SOX9* promoter region abundance in chromatin immunoprecipitation (ChIP) of endogenous PML protein using an antibody against PML in MDA-MB-231 cells (n=4). **(h-i)** *SOX9* gene expression (n=8) **(h)** and representative Western blot (out of three independent experiments) showing PML and SOX9 expression **(i)** upon 6 days of ectopic PML induction with doxycycline treatment (50 ng/ml). **(j)** SOX9 DNA region immunoprecipitated with PML according to ENCODE data set. Italics-underlined nucleotides indicate primer chosen to sequence this region and bold-underlined nucleotides indicate de sequencing results from the HA-PML ChIP-qPCR band. Error bars represent s.e.m. p, p-value (*p< 0.05, **p< 0.01, ***p< 0.001 compared with control). Statistics test: one tail unpaired T test (a-e, g, h). shC: Scramble shRNA, sh2, sh4 and sh5: shRNA against *PML*, VC: vehicle control, ATO: arsenic trioxide, dox: doxycycline.

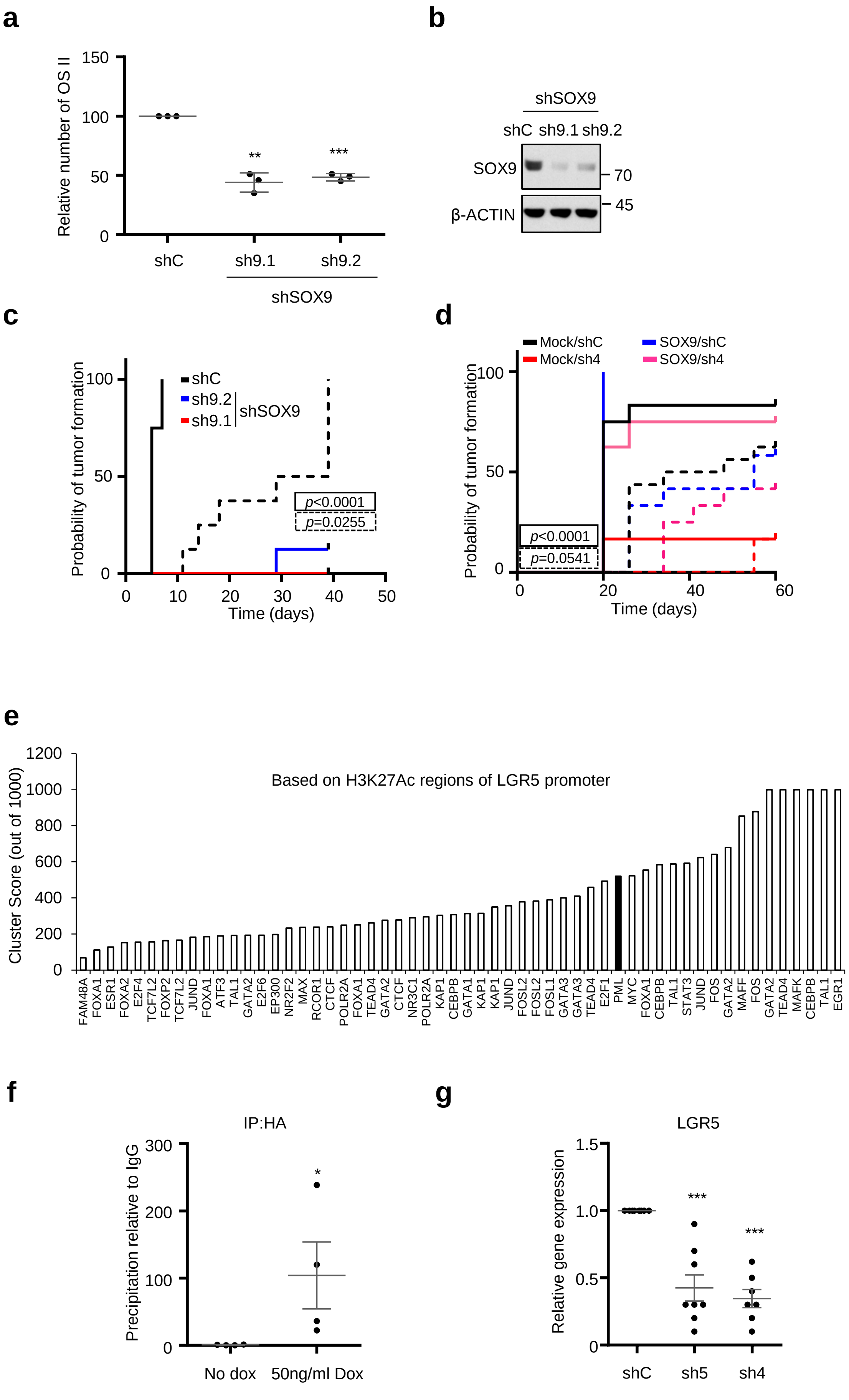

Supplementary Figure 6. Extended data related to Figure 6.

(a) Effect of *SOX9* silencing with 2 different shRNAs (sh1 and sh2) on OSII formation in MDA-MB-231 cells (n=3). **(b)** Representative Western blot of SOX9 expression at the time of injection in (c). **(c)** Kaplan-Meier curves of tumour formation after inoculation with 500.000 (solid line) or 50.000 (dashed line) MDA-MB-231 cells transduced with sh1 and sh2 against *SOX9* (n=8 injections per experimental condition). **(d)** Kaplan-Meier curves of tumour formation after inoculation with 500.000 (solid line) or 50.000 (dashed line) MDA-MB-231 cells transduced with the indicated constructs (Injections per experimental condition: 500.000 cells shC cells=12; 500.000 cells shPML4=16; 50.000 cells shC+Mock=16; 50.000 cells shC+SOX9/sh4+Mock/sh4+SOX9=12). **(e)** Cluster score of DNA binding proteins in *LGR5* promoter region using ENCODE database. **(f)** *LGR5* promoter region abundance in chromatin immunoprecipitation (ChIP) of exogenous HA-PMLIV using HA-tag antibody in MDA-MB-231 cells after induction with 50 ng/ml doxycycline during 3 days (n=4). Data were normalized to IgG (negative binding control). **(g)** *LGR5* gene expression upon *PML* silencing with 2 different shRNAs in MDA-MB-231 cells (sh4 n=7, sh5 n=8). Error bars represent s.e.m. p, p-value (*p< 0.05, **p< 0.01, ***p< 0.001 compared with control). Statistics test: one tail unpaired T test (a, f, g) and Log-rank (Mantel-Cox) test (c, d). shC: Scramble shRNA, sh9.1 and sh9.2: shRNA against *SOX9*, sh4, sh5: shRNA against *PML*, OSII: secondary oncospheres, dox: doxycycline.

Figure 1a

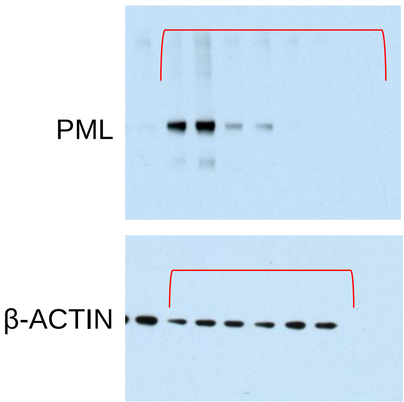

Figure 1e

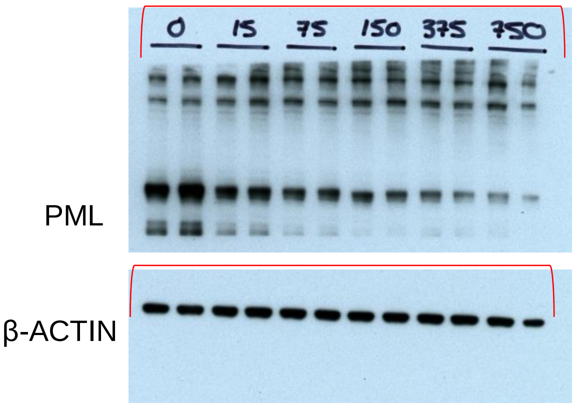

Figure 1i

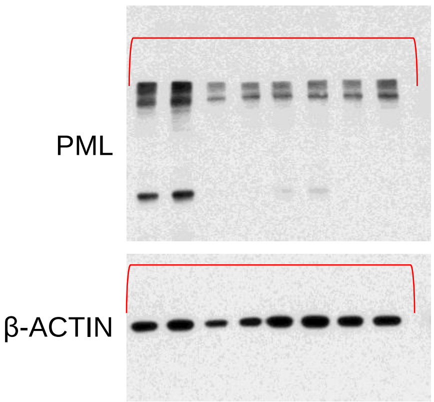

Supplementary Figure 1c

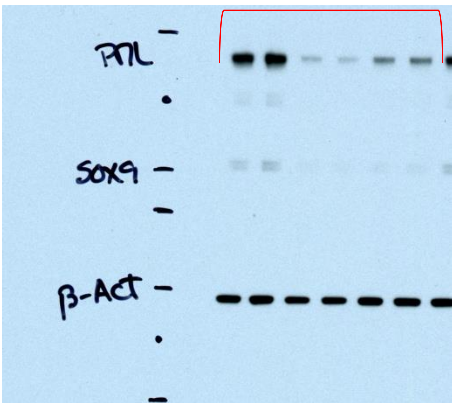

Figure 2c

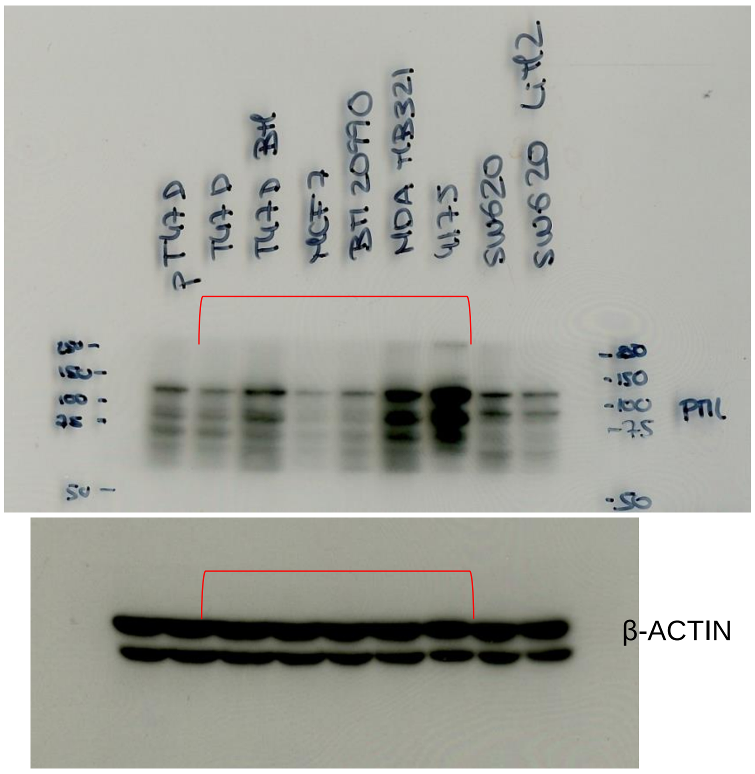

Figure 2d

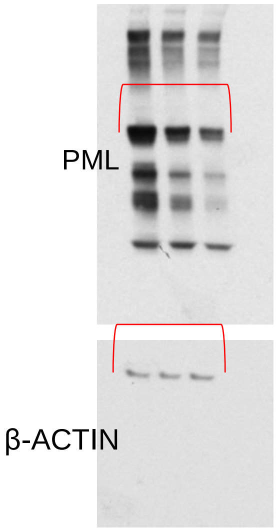

Supplementary Figure 7. Uncropped scans.

Figure 3a (inverted for clarity)

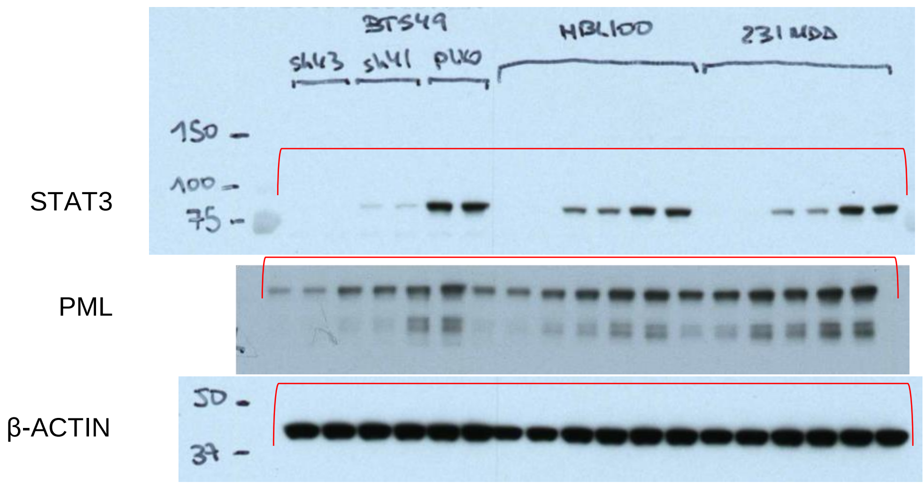

Figure 3b

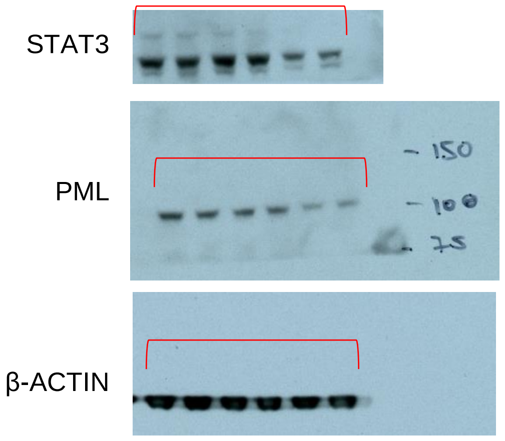

Figure 3c (inverted for clarity)

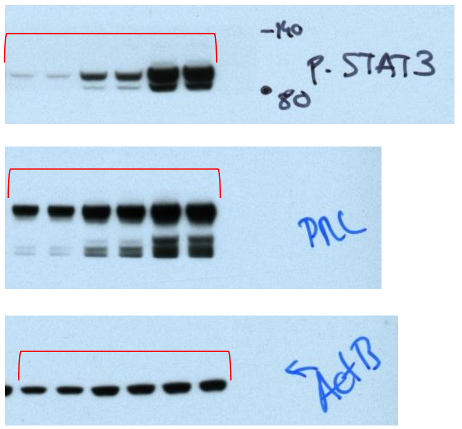

Figure 4a

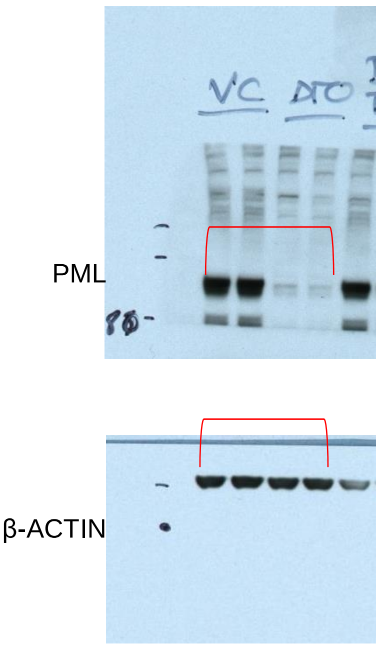

Figure 4b

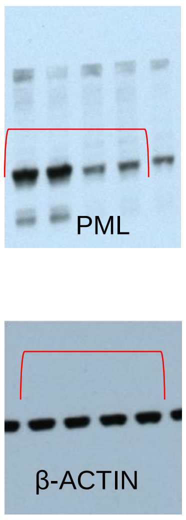

Figure 4c

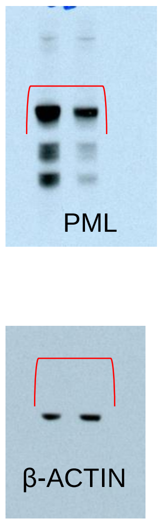

Figure 4d

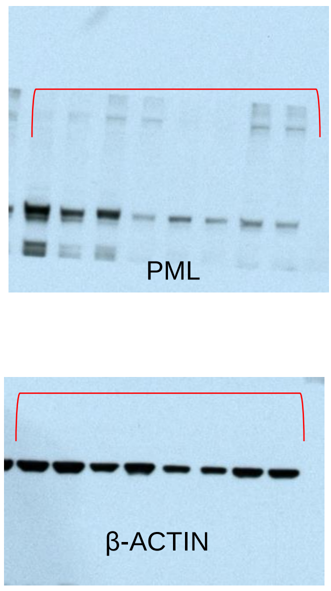

Figure 4e

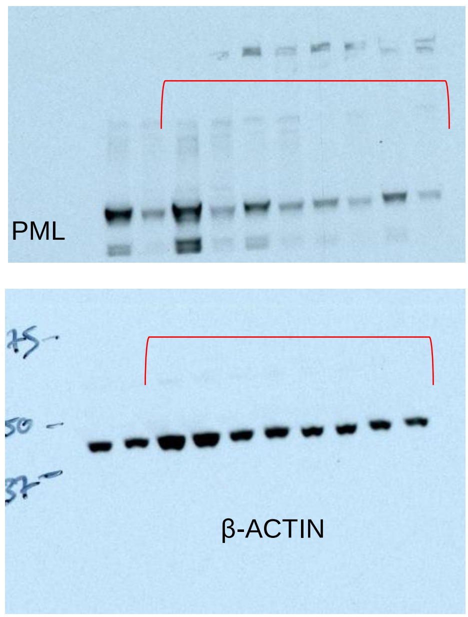

Figure 4g

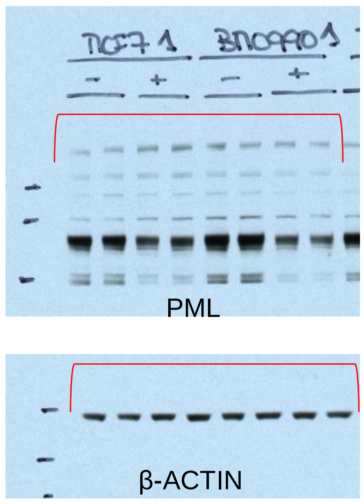

Supplementary Figure 7 cont. Uncropped scans.

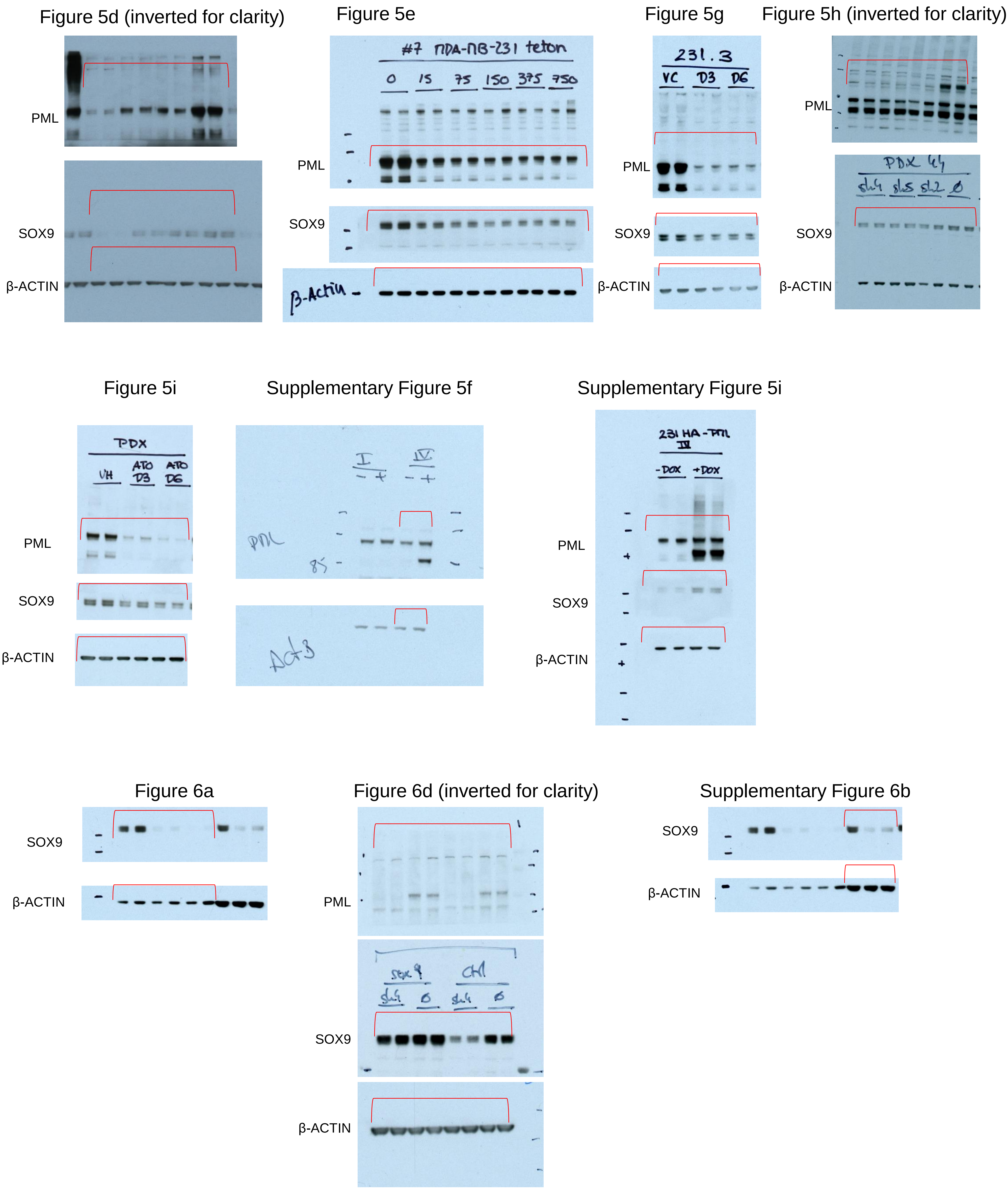

Supplementary Figure 7 cont. Uncropped scans.
